# IL-22 promotes the establishment of chronic gammaherpesvirus infection by supporting the germinal center response

**DOI:** 10.1371/journal.ppat.1014016

**Published:** 2026-09-08

**Authors:** Sheikh Tahir Majeed, Samantha M. Bradford, Christopher N. Jondle

**Affiliations:** Department of Investigative Medicine and Center for Immunobiology, Western Michigan University Homer Stryker M.D. School of Medicine, Kalamazoo, Michigan, United States of America; Wistar Institute, UNITED STATES OF AMERICA

## Abstract

Gammaherpesviruses, including human Epstein-Barr Virus (EBV) and Kaposi’s Sarcoma-associated Herpesvirus (KSHV), establish lifelong latent infections that contribute to multiple cancers and Multiple Sclerosis. These viruses colonize naïve B cells and drive a robust, polyclonal germinal center response to expand the latent viral reservoir and establish lifelong infection in memory B cells. Despite the clinical burden of these viruses, the host factors that support chronic infection remain poorly defined. Interleukin-22 (IL-22) is a critical cytokine traditionally recognized for its protective roles in bacterial and fungal defense; however, its involvement in gammaherpesvirus pathogenesis has not been explored. Using murine gammaherpesvirus 68 (MHV68) as a tractable *in vivo* model, we identify IL-22 as a host factor that promotes the establishment of chronic gammaherpesvirus infection. MHV68 infection triggers robust IL-22 production across multiple anatomical sites and immune cell populations. Although IL-22 deficiency did not affect acute viral replication, IL-22^-/-^ mice exhibited a sustained reduction in latent viral burden within both the spleen and peritoneal cavity. This phenotype was accompanied by impaired germinal center B cell and T follicular helper cell responses, reduced plasma-cell expansion, diminished virus-specific antibody responses, and attenuated polyclonal autoreactive antibody production. Furthermore, IL-22 deficiency was associated with reduced systemic BAFF levels, suggesting that IL-22 contributes to the establishment of a germinal center microenvironment that supports chronic infection. Together, these findings identify IL-22 as a previously unrecognized regulator of chronic gammaherpesvirus infection and reveal a role for this cytokine in coordinating germinal center responses, humoral immunity, and latent reservoir establishment, providing new insight into how host immune pathways contribute to lifelong gammaherpesvirus infection.

## Author summary

Gammaherpesviruses are double-stranded DNA viruses that establish lifelong infections in over 95% of adults worldwide and are associated with multiple cancers and autoimmune disease. To persist within the host, these viruses exploit germinal center B cells to establish a long-lived latent reservoir. Although the cytokine IL-22 is best known for its protective role in bacterial and fungal infections, its contribution to gammaherpesvirus infection was unknown. Using a murine model of gammaherpesvirus infection, we demonstrate that IL-22 promotes the establishment of chronic gammaherpesvirus infection without affecting acute viral replication. Mice lacking IL-22 exhibited reduced latent viral burden accompanied by impaired germinal center and plasma-cell responses along with diminished virus-specific and polyclonal antibody production. These findings suggest that IL-22 helps create an immune environment that favors viral latency. Our work identifies a previously unrecognized role for IL-22 in supporting chronic gammaherpesvirus infection and provides new insights into how host immune pathways contribute to lifelong gammaherpesvirus infection.

## Introduction

Gammaherpesviruses, including human Epstein-Barr virus (EBV) and Kaposi’s sarcoma-associated herpesvirus (KSHV), are ubiquitous pathogens that infect over 95% of the adult population worldwide and establish lifelong infection in their host [1–3]. They are etiological agents for a broad spectrum of malignancies, including cancers such as Burkitt’s lymphoma, Hodgkin’s lymphoma, and Kaposi’s sarcoma, particularly in immunocompromised individuals. Beyond oncology, chronic gammaherpesvirus infection has been increasingly linked to the development of systemic autoimmune diseases, most notably Multiple Sclerosis [4]. The lifecycle of these viruses is characteristically biphasic, comprising an initial lytic replication phase (acute) followed by the establishment of viral latency (chronic phase) [5,6]. Infection typically initiates in mucosal epithelial cells and macrophages in lungs before disseminating to secondary lymphoid tissues, such as the spleen and peritoneal cavity [7,8]. Once in these compartments, the virus colonizes naïve B cells and drives a robust germinal center response [9,10]. This expansion of B cells is critical for the viral life cycle, allowing the virus to differentiate into memory B cells, the primary reservoir for lifelong latency, or plasma cells, from which the virus will reactivate [11]. Notably, the high proliferative rate of germinal center B cells, combined with the downregulation of tumor suppressors, makes this niche a primary site for cellular transformation, leading to the high incidence of germinal center-derived B-cell lymphomas associated with these infections [12–15]. Despite their clinical significance, the host factors that facilitate the establishment of these chronic infections remain incompletely understood, largely due to the extreme species-specificity of human gammaherpesviruses which limits *in vivo* mechanistic studies. To overcome these limitations, murine gammaherpesvirus 68 (MHV68) is utilized as a tractable and biologically relevant animal model [5,6]. MHV68 shares significant genetic and pathogenetic homology with its human counterparts, including the ability to replicate in lung epithelium before establishing a robust latent reservoir in germinal center B cells [5,16–18]. In this model, the establishment of latency is a highly orchestrated process where the virus usurps host immune signaling to promote its own latent reservoir. Among the host factors potentially involved in this process, interleukin-22 (IL-22) emerges as a candidate of significant interest due to its potent regulatory effects on mucosal barriers and lymphoid architecture [19,20].

Interleukin-22 (IL-22), originally named as IL-10-related T cell-derived inducible factor (IL-TIF), is a member of IL-10 family of cytokines [21,22]. IL-22 is expressed by various immune cell subsets, including activated natural killer (NK) cells, NKT cells, neutrophils, innate lymphoid cells (ILCs), γδ T cells, CD8 + T cells [23–25], and CD4 + T cell subsets including of the TH17 [26,27], TH1 [28] and TH22 [29]. Uniquely, IL-22 signaling is directed toward non-hematopoietic cells through a heterodimeric receptor complex (IL-22Rα and IL-10Rβ). IL-10Rβ is broadly expressed by most cell types, whereas the expression of IL-22Rα is restricted to non-hematopoietic cells [27,30]. IL-22 downstream signaling leads to the activation of Jak/STAT pathway—primarily STAT3—and MAP kinase/p38 pathways [30,31]. While IL-22 has a well-defined role in clearing bacterial and fungal pathogens, its role in viral infections is increasingly recognized as complex and context-dependent.

Emerging reports suggest that IL-22 can be either host-protective or proviral. In Rotavirus and MCMV infections, IL-22 exerts antiviral effects through cooperation with other cytokines or the recruitment of neutrophils [32,33]. Conversely, in West Nile Virus and Hepatitis B models, IL-22 can exacerbate tissue pathology or support virus-driven inflammation. This is achieved by inducing the expression of pro-inflammatory chemokines such as CXCL1, CXCL5, and CXCL10, which promotes the excessive recruitment of neutrophils and other inflammatory infiltrates into infected tissues [34,35]. Furthermore, EBV-induced infectious mononucleosis has been shown to elevate serum IL-22 levels in both adults and children [36,37]. Despite these insights, the specific role of host IL-22 in orchestrating the gammaherpesvirus life cycle and the establishment of latency remains unknown.

Our previous work identified a proviral role for the IL-17RA signaling axis during the establishment of chronic MHV68 infection [38–40]. Given that IL-22 and IL-17 are frequently co-expressed and cooperate to regulate tissue homeostasis and antimicrobial responses [41], we hypothesized that IL-22 might similarly influence chronic MHV68 infection. However, despite this functional overlap, IL-17 and IL-22 are likely to promote chronic infection through distinct mechanisms. Whereas IL-17RA is broadly expressed on hematopoietic and non-hematopoietic cell populations [42–44], IL-22 signaling is largely restricted to non-hematopoietic cells through IL-22Rα [27,30]. Thus, although both cytokines may ultimately promote viral latency, they are likely to influence different cellular compartments and biological processes within the infected host. Notably, because gammaherpesviruses do not encode a discernible IL-22 homolog, they must rely on modulation of host-derived IL-22 to shape the immune microenvironment that supports establishment of chronic infection. In this study, we investigated the role of host IL-22 during MHV68 infection. We demonstrate that MHV68 induces a robust IL-22 response across multiple tissues and immune cell populations. Although IL-22 deficiency does not impair acute viral replication, it results in a reduced latent viral reservoir accompanied by diminished germinal center B cells and T follicular helper cell responses, impaired plasma-cell expansion, as well as defective virus-specific and polyclonal antibody responses. Collectively, our findings identify IL-22 as a previously unrecognized host pathway that supports the establishment of chronic gammaherpesvirus infection and reveal a role for IL-22 in coordinating the germinal center and humoral responses that accompany viral infection.

## Results

### MHV68 infection elicits IL-22 expression across multiple anatomic sites and immune cell populations

Due to the extreme species-specificity of human gammaherpesviruses EBV and KSHV, *in vivo* studies of their host interactions are inherently limited. To address this, we utilized the genetically and biologically related murine gammaherpesvirus 68 (MHV68), an established and tractable model for studying gammaherpesvirus pathogenesis in an immunocompetent host [5,45–47].

**Fig 1:**
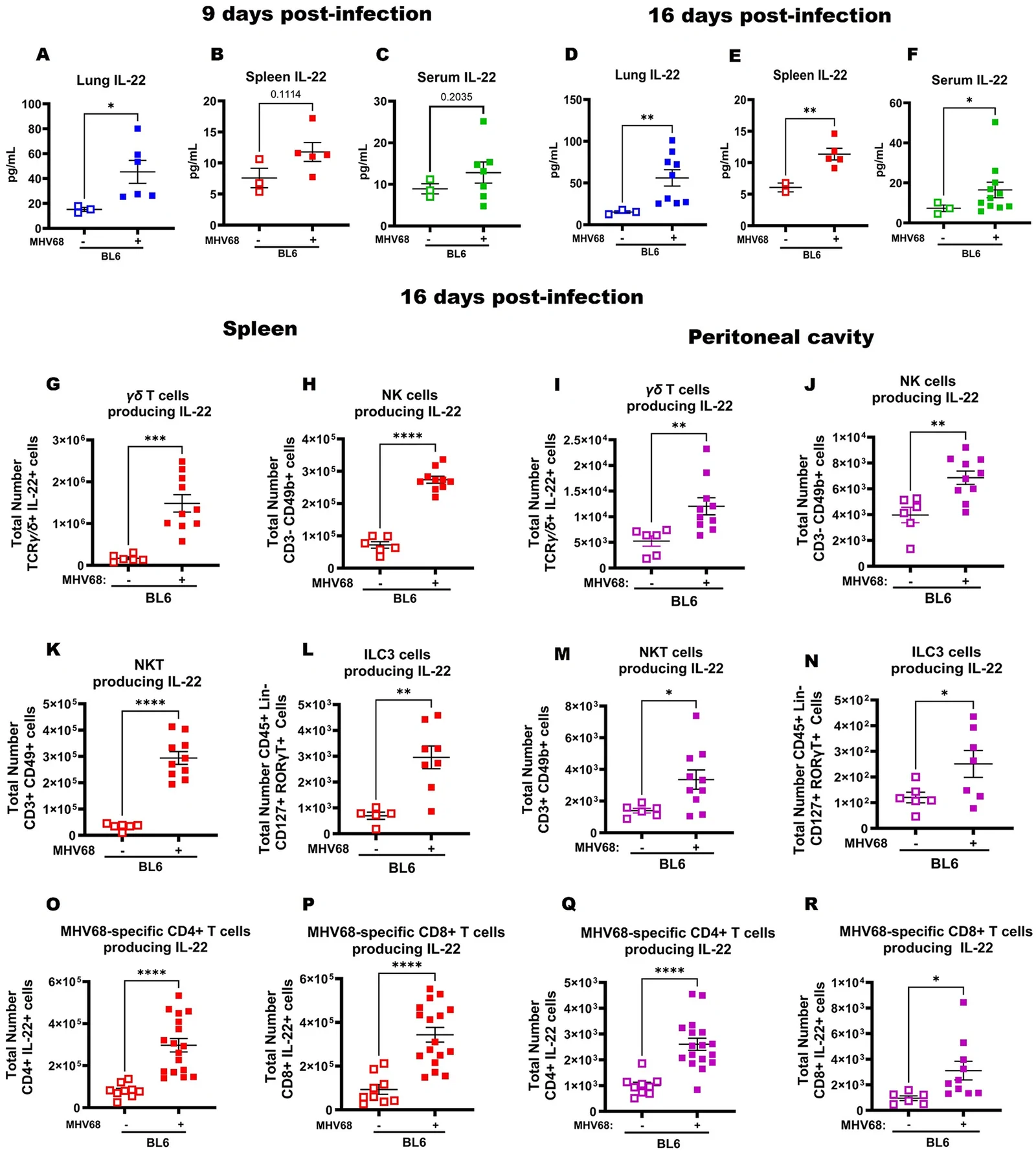
MHV68 infection induces IL-22 production across anatomical sites and immune cell populations. C57BL/6 (BL6) mice were intranasally infected with 1000 PFU of MHV68. Tissues and serum were harvested at 9 dpi and 16 dpi, corresponding to the lytic and peak latent phases of infection, respectively. **(A–D)** IL-22 protein levels in the lungs at 9 dpi (**A**) and 16 dpi (**D**) were determined by ELISA. **(B, E)** IL-22 protein levels in the spleens at 9 dpi (**B**) and 16 dpi (**E**) were quantified by ELISA. **(C, F)** Serum IL-22 concentrations at 9 dpi (**C**) and 16 dpi (**F**) were measured using a flow cytometry-based multiplex bead assay. **(G–J)** Total numbers of IL-22-producing γδ T cells (defined as TCRγδ + IL-22 + cells) (**G, I**), NK cells (defined as CD3-CD49 + IL-22 + cells) **(H,J)**, NK T cells (defined as CD3 + CD49 + IL-22 + cells) **(K,M)**, and ILC3 cells (defined as CD45 + Lineage-Thy1 + TCRβ- TCRγδ-CD127 + RORγT + IL-22 + cells) (**L,N**) in the spleen and peritoneal cavity, respectively, at 16 dpi. **(K–N)**. **(O–R)** Total numbers of IL-22-producing virus-specific CD4⁺ (defined asCD3 + CD4 + IL-22 + cells) (**O,Q**) and CD8⁺ (defined as CD3 + CD8 + IL-22 + cells) **(P,R)** T cells in the spleen and peritoneal cavity, respectively, at 16 dpi. Virus-specific T cells were identified following ex vivo stimulation with the immunodominant MHV68 peptides gp150 (MHC class II) or ORF6 (MHC class **I)**, followed by intracellular cytokine staining and flow cytometric analysis. Each experimental group consists of 3–4 animals and each symbol represent an individual mouse. Data are pooled from two to three independent experiments. Mean and standard error of the mean are shown. *P < 0.05, **P < 0.01, ***P < 0.001, ****P < 0.0001.

Following intranasal MHV68 infection, we characterized host IL-22 expression patterns in the lungs and spleen, two primary sites of viral infection. We examined C57BL/6 wild-type (BL6) mice at 9 days post-infection (dpi) and 16 dpi, representing lytic (acute) and peak of latent (chronic) infection, respectively. At 9 dpi, IL-22 levels showed significant increase in lungs (Fig 1A), while as the spleen exhibited increasing trend in the levels of IL-22 without reaching any statistical significance (Fig 1B). Importantly, while the onset of latent infection at 16 dpi continued to show significant levels of pulmonary IL-22 levels, its expression levels in the spleen reached to significant levels at this time point (Fig 1D, E). Furthermore, systemic IL-22 levels in the serum of MHV68-infected BL6 mice were also trending higher at 9 dpi and reached significant levels at 16 dpi (Fig 1C, F), indicating that MHV68 infection induces both local and systemic IL-22 responses.

Given that IL-22 is produced by a broad range of innate and adaptive immune cell populations [48], we next sought to identify cellular sources contributing to this response. We quantified IL-22-producing immune cell subsets within the spleen and peritoneal cavity at 16 dpi. MHV68 infection significantly increased the total numbers of IL-22-producing γδ T cells, natural killer (NK) cells, natural killer T (NKT) cells, and group 3 innate lymphoid cells (ILC3s) in both compartments (Fig 1G–Fig 1N) [49]. In addition to these populations, virus-specific adaptive immune responses also contributed substantially to the IL-22 pool. Following *ex vivo* restimulation with the immunodominant MHV68 peptides gp150 (MHC class II) and ORF6 (MHC class I), we observed a significant increase in the total numbers of IL-22-expressing, virus-specific CD4+ and CD8 + T cells in both these compartments (Fig 1O-Fig 1R, Fig 1K). Collectively, these data demonstrate that MHV68 infection elicits a robust IL-22 response across multiple anatomic locations and that both innate and adaptive immune cell populations contribute to the increased IL-22 production observed during chronic infection.

### IL-22 deficiency does not affect acute MHV68 replication

Following inoculation of a naïve host, MHV68 undergoes lytic replication during the first 7–12 days post-infection (dpi). During this acute phase, the virus predominantly infects lung epithelial cells and macrophages before subsequently disseminating to lymphoid tissues and establishing latency within B cells [50,51]. Given the robust induction of IL-22 during MHV68 infection (Fig 1), we next investigated whether IL-22 contributes to acute viral replication.

**Fig 2:**
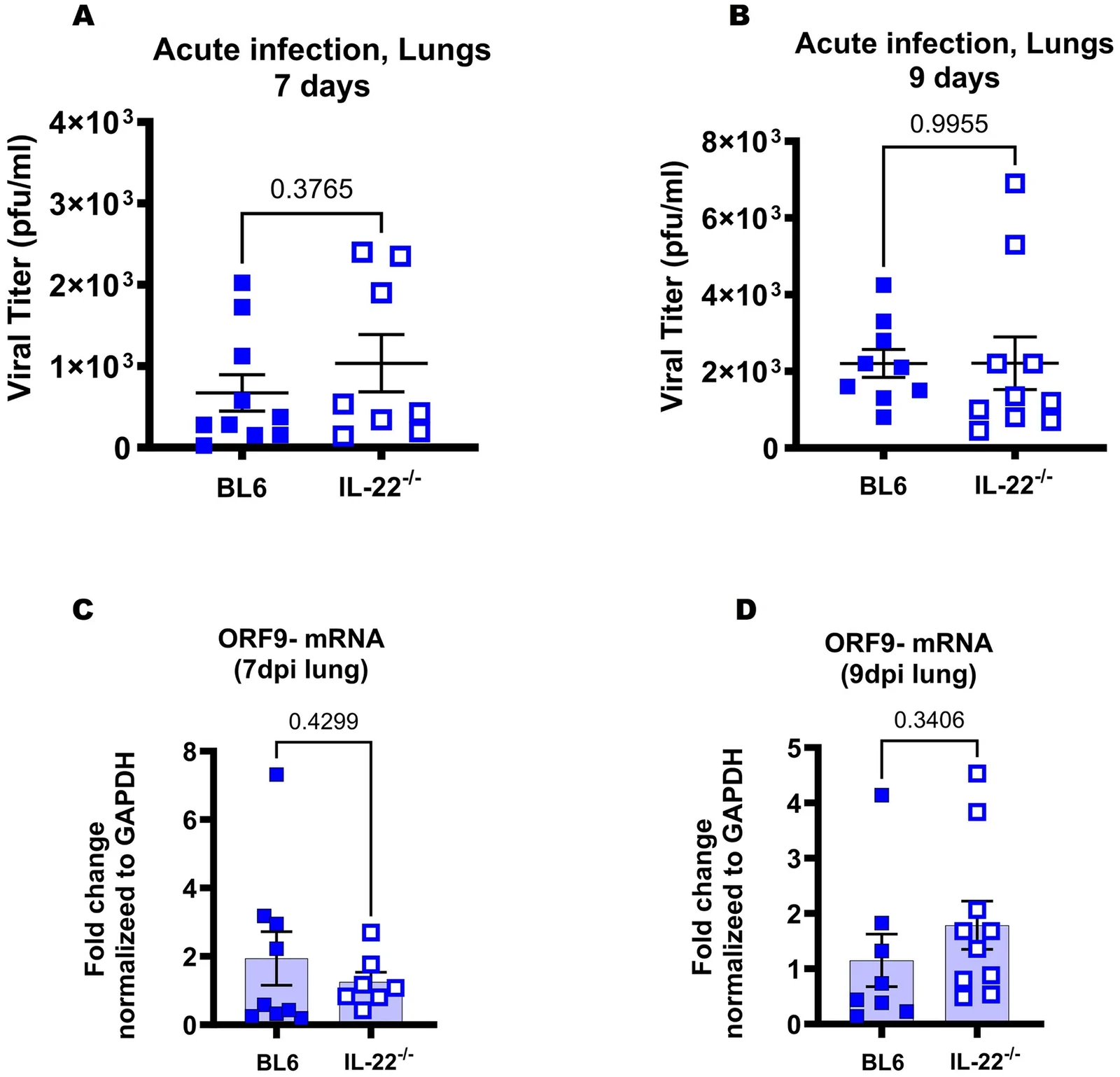
IL-22 deficiency does not impair acute MHV68 replication. BL6 and IL-22^-/-^ mice were infected as described in the legend to Fig 1 and analyzed during the acute phase of infection. **(A-B)** Lung viral titers at 7 days post-infection (dpi) (**A**) and 9 dpi (**B**) were determined by plaque assay. **(C-D)** Relative expression of the lytic viral gene *ORF9* in lungs harvested at 7 dpi (**C**) and 9 dpi (**D**) was determined by quantitative RT-PCR and normalized to GAPDH expression. Data are pooled from two independent experiments. Each experimental group consists of 4–5 animals and each symbol represent an individual mouse. Mean and standard error of the mean are shown.

To assess peak lytic infection, pulmonary viral titers were quantified at 7 dpi. Consistent with the established tropism of MHV68 during acute infection, viral loads in the lungs were comparable between BL6 and IL-22^-/-^ mice (Fig 2A). We next examined viral burden at 9 dpi, a time point corresponding to the initiation of viral clearance. Similar to our observations at 7 dpi, lung viral titers remained indistinguishable between the two genotypes (Fig 2B).

To independently confirm these findings, we measured expression of the lytic viral gene ORF9 within infected lung tissue. Consistent with the viral titer data, ORF9 transcript levels were equivalent between BL6 and IL-22^-/-^ mice at both 7 and 9 dpi (Fig 2C, D), indicating that loss of IL-22 does not impair lytic viral gene expression during acute infection.

Collectively, these findings demonstrate that IL-22 is dispensable for acute MHV68 replication in the lung, suggesting that IL-22 does not significantly influence lytic infection.

### IL-22 expression supports gammaherpesvirus latency and ex-vivo reactivation

Having established that MHV68 infection induces a robust IL-22 response (Fig 1) while IL-22 deficiency does not affect acute viral replication (Fig 2), we next investigated whether IL-22 influences the establishment of chronic infection. BL6 and IL-22-deficient (IL-22^-/-^) mice were infected intranasally with MHV68 and latency parameters were evaluated at the peak of viral latency (16 dpi), within the spleen and peritoneal cavity, the two principal reservoirs of latent MHV68 [5].

**Fig 3:**
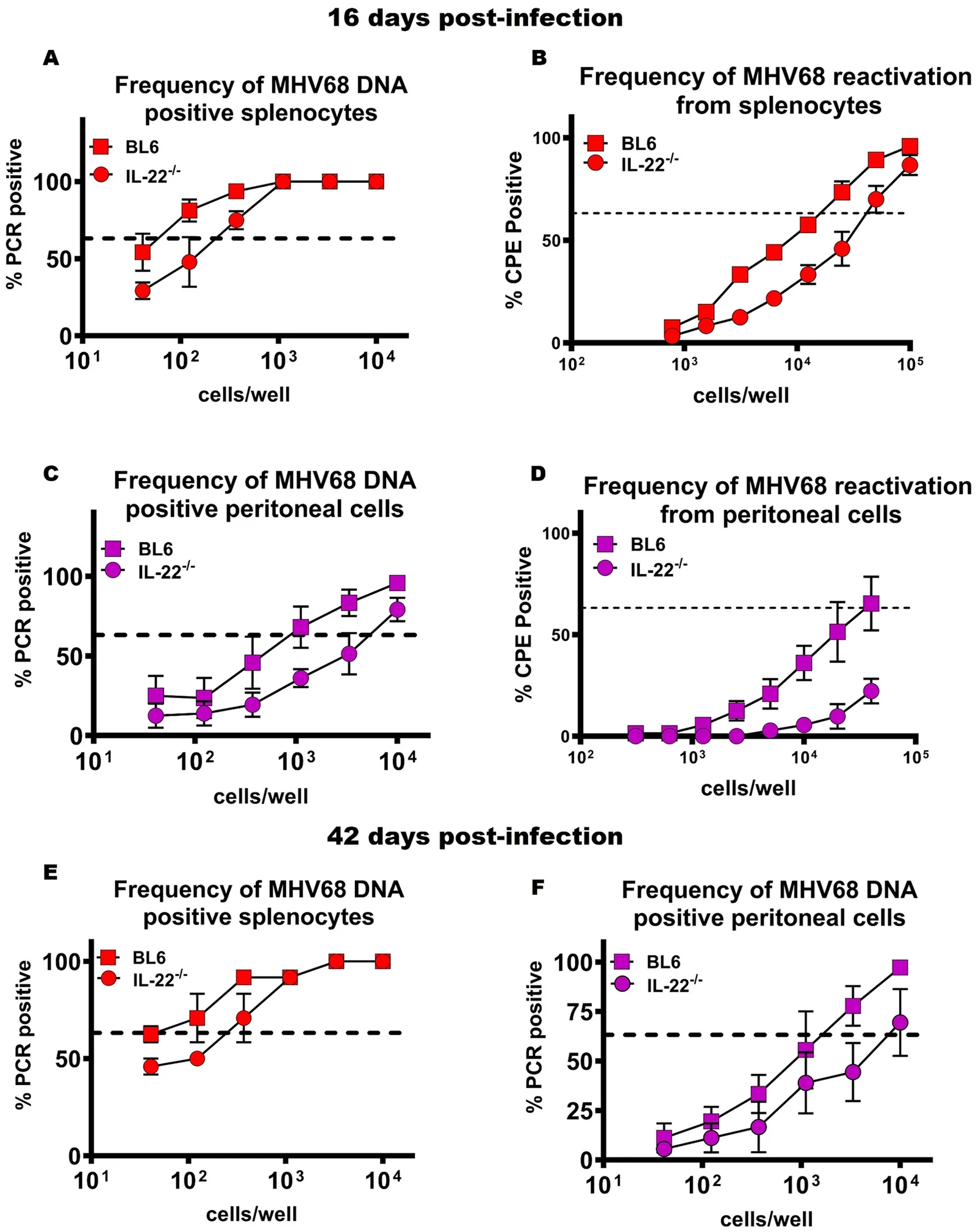
IL-22 deficiency reduces latent viral reservoir and *ex vivo* reactivation. BL6 and IL-22^-/-^ mice were infected as described in the legend to Fig 1. Splenocytes and peritoneal cells were harvested at 16 dpi (peak latency) and 42 dpi (long-term latency). **(A, C)** The frequency of MHV68 DNA-positive cells in the spleen (**A**) and peritoneal cavity (**C**) at 16 dpi were determined by limiting-dilution PCR (LD-PCR). **(B, D)** The frequency of *ex vivo* viral reactivation from splenocytes (**B**) and peritoneal cells (**D**) at 16 dpi. Cells were serially diluted onto MEF monolayers and monitored for cytopathic effect (CPE) at 21 days post-challenge. **(E, F)** The frequency of MHV68 DNA-positive cells in the spleen (**E**) and peritoneal cavity (**F**) during long-term infection (42 dpi) were determined by LD-PCR. Each experimental group consists of three to five animals. Data were pooled from two to five independent experiments, and SEM is displayed. In the limiting dilution assays, the dotted line is drawn at 63.2%, and the x-coordinate of the intersection of this line with the sigmoid graph represents the inverse of the frequency of positive events.

To quantify the latent viral load, we performed limiting-dilution PCR (LD-PCR) analysis to determine the frequency of MHV68 DNA-positive cells. Compared to infected BL6 controls, IL-22^-/-^ mice exhibited a reduction in the frequency of MHV68 DNA-positive cells in both the spleen and the peritoneal cavity at 16 dpi (Fig 3A, C; frequencies of the viral latency shown in Table 1). Since gammaherpesviruses can periodically reactivate from the latent phase [52], we next assessed the impact of IL-22 deficiency on viral reactivation competency via limiting dilution assay (LDA). Explanted splenocytes and peritoneal cells were serially diluted onto mouse embryonic fibroblast (MEF) monolayers and monitored for cytopathic effects (CPE) at 21 days post-plating. Consistent with the reduction in DNA-positive cells, the frequency of *ex-vivo* MHV68 reactivation was attenuated in the spleen and peritoneal cavity of IL-22^-/-^ mice compared to their BL6 counterparts at 16 dpi (Fig 3B, D; frequencies of the viral ex-vivo reactivation shown in Table 1). Together, these findings indicate that IL-22 signaling promotes viral reactivation and the establishment of MHV68 latency.

**Table 1:** Frequency of latency and reactivation.

Mouse strain	Cell population	dpi ^a^	Avg frequency of MHV68 genome-positive cells ^b^
BL6	Splenocytes	16	1/55
IL-22^-/-^	Splenocytes	16	1/208
BL6	Peritoneal cells	16	1/945
IL-22^-/-^	Peritoneal cells	16	1/4882
BL6	Splenocytes	42	1/49
IL-22^-/-^	Splenocytes	42	1/230
BL6	Peritoneal cells	42	1/1,550
IL-22^-/-^	Peritoneal cells	42	1/7,460
Mouse strain	**Cell population**	**dpi** ^**a**^	**Avg frequency of MHV68 reactivation** ^**c**^
BL6	Splenocytes	16	1/3,800
IL-22^-/-^	Splenocytes	16	1/12,250
BL6	Peritoneal cells	16	1/8,300
IL-22^-/-^	Peritoneal cells	16	>1/40,000

^a^dpi, days postinfection.

^b^Average frequency of cells harboring MHV68 genomes. The average frequencies derived from two to six independent experiments are shown.

^c^Average frequency of cells exhibiting ex-vivo reactivation of MHV68. The average frequencies derived from three to five independent experiments are shown.

Following the peak of latency, MHV68 infection transitions into a stable long-term phase. By 42 dpi, the latent reservoir reaches a relatively constant set-point that is maintained throughout the life of the host, while reactivation typically declines to levels below the limit of detection [5]. To determine whether IL-22 supports the maintenance of chronic infection, we quantified viral latency at 42 dpi. Similar to the phenotype observed during peak latency, IL-22 deficiency resulted in a sustained reduction in the frequency of MHV68 DNA-positive cells in the spleen and peritoneal cavity at 42 dpi (Fig 3E, F; frequencies of the viral latency shown in Table 1). As expected, no detectable reactivation was observed in either IL-22^-/-^ or BL6 mice at this late time point. Collectively, these findings suggest that IL-22 supports both the establishment of peak latency and the long-term maintenance of the latent viral reservoir.

### Loss of IL-22 attenuates virus-specific CD8 ⁺ T Cell expansion during chronic MHV68 infection

**Fig 4:**
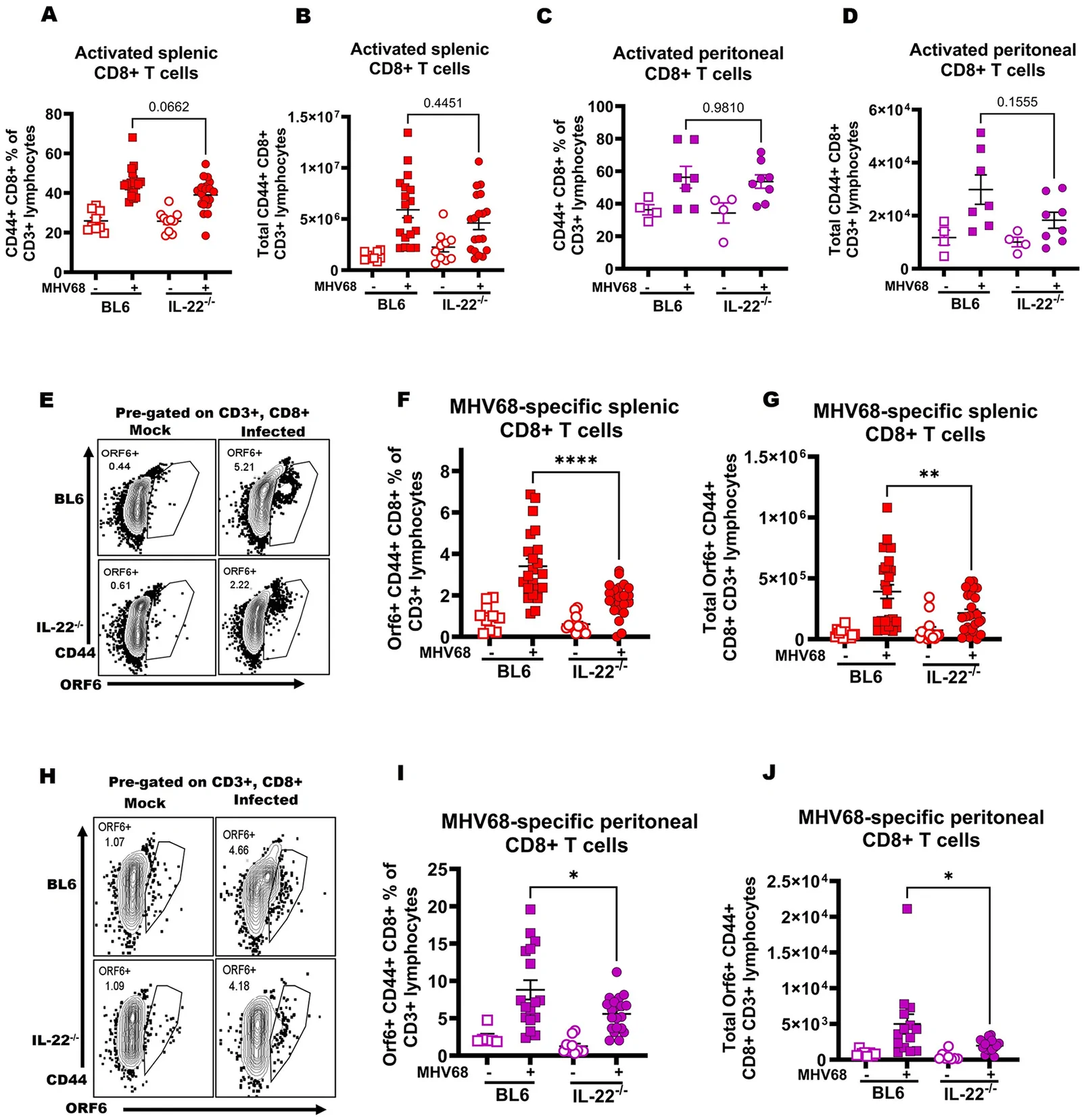
IL-22 deficiency attenuates virus-specific CD8 + T cell responses during chronic MHV68 infection. BL6 and IL-22^-/-^ mice were infected as described in the legend to Fig 1. CD8 + T cells were assessed at 16 dpi (peak latency) in the spleen and peritoneal cavity (PEC). **(A–D)** Frequency and absolute numbers of activated CD8 + T cells (defined as CD3 + CD8 + CD44 + cells) in the spleen (**A, B**) and PEC (**C, D**) were determined by flow cytometry. **(E-J)** Frequency and absolute number of MHV68-specific CD8 + T cells (defined CD3 + CD8 + CD44 + ORF6+) in the spleen (**E-G**) and peritoneal cavity **(H-J)**, identified via MHC-I tetramer staining for the immunodominant ORF6_487-495_ (p56) epitope were determined by flowcytometry. Each experimental group consists of 3–4 animals and each symbol represent an individual mouse. Data are pooled from two to five independent experiments. Mean and standard error of the mean are shown. *P < 0.05, **P < 0.01, ****P < 0.0001.

Having observed a significant reduction in viral latency and reactivation in MHV68-infected IL22^-/-^ mice at the peak of latency (Fig 3), we next investigated whether this diminished viral burden influenced the host immune response. CD8 + T cells play a critical role in controlling chronic gammaherpesvirus infection and limiting viral reactivation. First, viral specific CD8 + T cells contract the initial expansion of infected B cells [53]. Second, they utilize perforin-mediated cytotoxicity and IFNγ production to suppress viral reactivation and maintain latency [54]. Therefore, we quantified the frequency and absolute numbers of activated and virus-specific CD8 + T cells in the spleen and peritoneal cavity of infected BL6 and IL-22^-/-^ mice during chronic infection. While global CD8 + T cell- activation remained comparable between genotypes in both tissues (Fig 4A-D), the frequency and absolute numbers of virus-specific CD8 + T cells were significantly reduced in the spleen (Fig 4E-G) and peritoneal cavity (Fig 4H-J) of IL-22^-/-^ mice. The reduction in virus-specific CD8 ⁺ T-cell responses across both anatomical sites parallels the diminished latent viral burden observed in IL-22^-/-^mice, suggesting that reduced antigen availability during chronic infection contributes to the blunted expansion of virus-specific CD8 ⁺ T cells. Together, these findings indicate that IL-22 deficiency selectively impairs the accumulation of virus-specific CD8 ⁺ T cells during chronic MHV68 infection without altering global CD8 ⁺ T-cell activation.

### IL-22 is not required for CD8+ T cell functional capacity or systemic inflammatory stability

**Fig 5:**
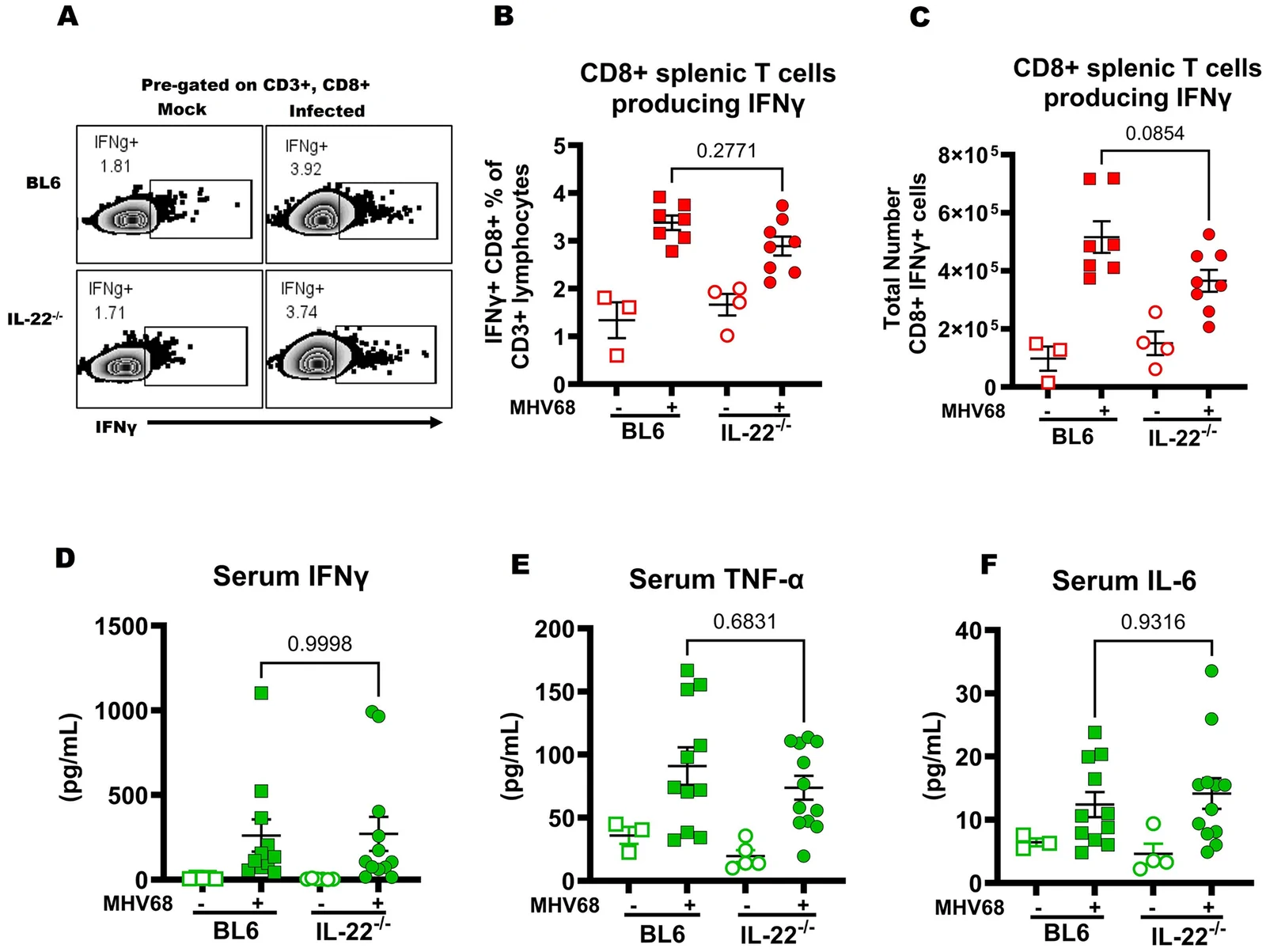
IL-22 is not required for CD8 + T cell functional capacity or systemic inflammatory stability. BL6 and IL-22^-/-^ mice were infected as described in the legend to Fig 1. **(A-C)** Flow cytometry-based analysis of IFNγ production by viral specific splenic CD8 + T cells at 16dpi. Splenocytes were restimulated *ex vivo* with Orf6 MHV68 peptide, and the frequency (**A, B**) and absolute number (**C**) of IFNγ+ cells were determined by intracellular cytokine staining (ICS). **(D–F)** Systemic inflammatory milieu at 16 dpi. Serum levels of IFNγ **(D)**, TNFα **(E)**, and IL-6 (**F**) were measured using a flow cytometry-based multiplex bead assay. Each experimental group consists of 3–4 animals and each symbol represent an individual mouse. Data are pooled from two to three independent experiments. Mean and standard error of the mean are shown.

Because IL-22 deficiency resulted in reduced virus-specific CD8 + T cell responses during chronic MHV68 infection, we next examined whether the functional capacity of these cells to produce gamma interferon (IFN-γ) was altered. Splenocytes were restimulated ex vivo with the immunodominant MHV68-derived peptide (ORF6_487–495_) and IFNγ production was assessed by intracellular cytokine staining. Despite the reduction in virus-specific CD8 ⁺ T-cell numbers observed in IL-22^-/-^ mice, both the frequency and absolute number of IFNγ-producing splenic CD8 ⁺ T cells were comparable between IL-22^-/-^ and BL6 mice (Fig 5A-C). We next assessed whether the loss of IL-22 altered the systemic inflammatory environment during chronic infection. Measurement of serum cytokines revealed no significant differences in circulating IFNγ, TNFα, or IL-6 levels between infected BL6 and IL-22^-/-^ mice (Fig 5D-F), indicating that the absence of IL-22 does not result in overt alterations in systemic inflammatory homeostasis during chronic infection. Collectively, these findings indicate that the reduced virus-specific CD8 ⁺ T-cell response observed in IL-22^-/-^ mice is not attributable to defects in splenic CD8 ⁺ T-cell effector function or disruptions in the systemic inflammatory milieu. Rather, it is consistent with diminished antigenic stimulation resulting from the reduced latent viral burden present in IL-22-deficient mice.

### IL-22 deficiency impairs germinal center expansion independent of global B cell activation and splenic pathology

**Fig 6:**
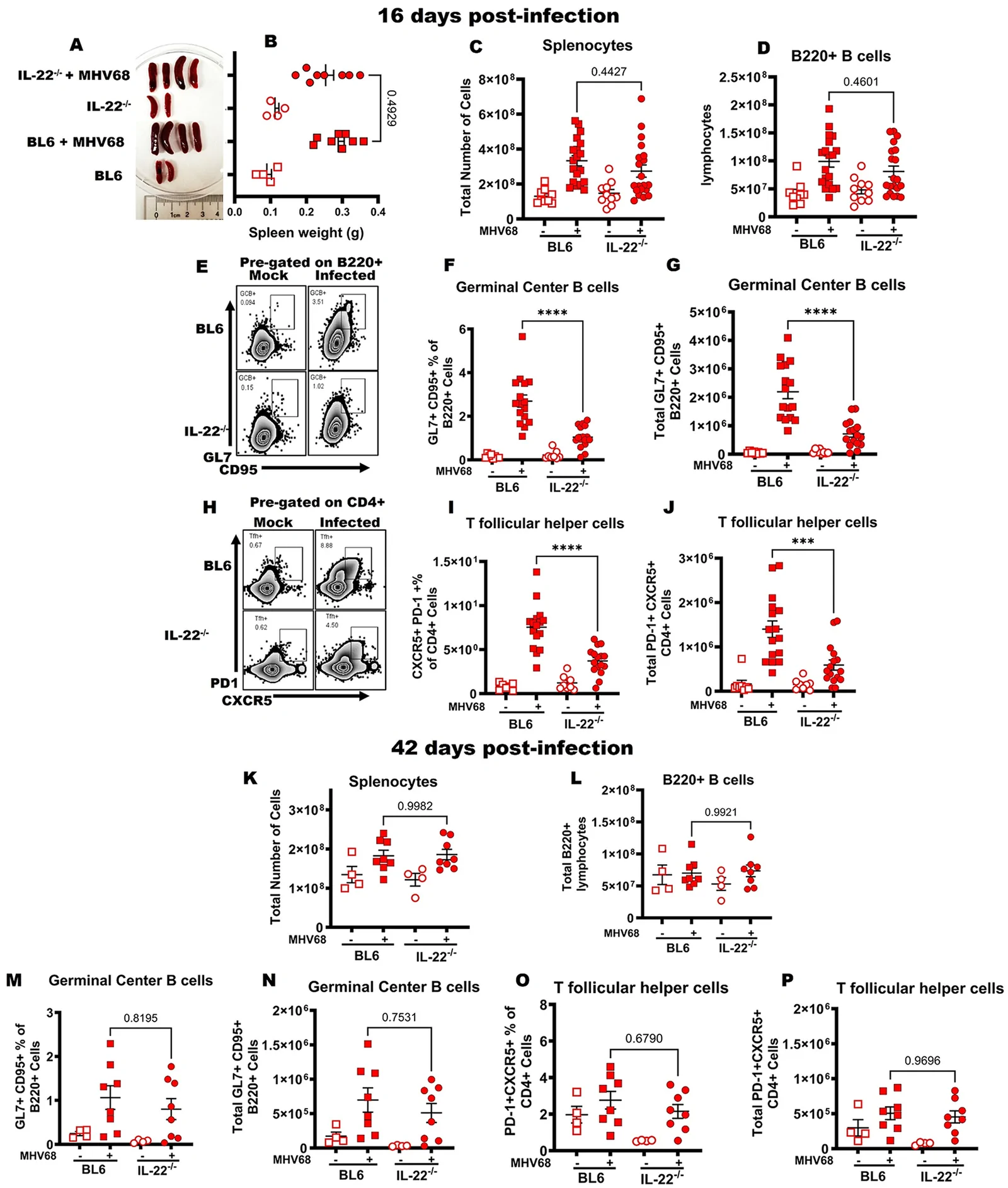
IL-22 deficiency impairs germinal center expansion independent of splenomegaly and global B cell accumulation. BL6 and IL-22^-/-^ mice were infected as described in Fig 1 and analyzed at 16 days post-infection (dpi), corresponding to the peak of latency. **(A-C)** Representative spleen morphology **(A)**, spleen weights **(B)**, and total splenocyte numbers (**C**) demonstrating comparable splenomegaly between infected BL6 and IL-22^-/-^ mice. **(D)** Total numbers of splenic B220 ⁺ B cells determined by flow cytometry. **(E-G)** representative flow cytometric plots **(G)**, frequencies (**F**) and total numbers (**G**) of germinal center (GC) B cells, defined as B220 ⁺ GL7 ⁺ CD95 ⁺ cells. **(H-J)** Representative flow cytometric plots (**H**) frequencies (**I**) and total numbers (**J**) of T follicular helper (Tfh) cells, defined as CD3 ⁺ CD4 ⁺ CXCR5 ⁺ PD-1 ⁺ cells **(K-L)** Total numbers of centroblasts (defined as CXCR4 + CD86-) (**K**) and centrocytes (defined as CXCR4-CD86+) (**L**) within the GC B-cell compartment. **(M-O)** Serum concentrations of BAFF **(M)**, CXCL12 **(N)**, and CXCL13 (**O**) were determined by flow cytometry-based multiplex bead assay. Each experimental group consists of 3–4 animals, and data are pooled from two to five independent experiments. Mean and standard error of the mean are shown. ***P < 0.001, ****P < 0.0001.

A characteristic feature of MHV68 infection is the induction of splenomegaly, driven by the lymphoproliferative expansion of both B and T cells [55]. Therefore, we next sought to determine if the loss of IL-22 altered the MHV68-induced splenic pathogenesis. Consistent with our findings that demonstrate intact global CD8 + T cell effector function and stable systemic cytokine levels (Fig 5), MHV68-infected IL-22^-/-^ mice developed gross splenomegaly comparable to infected BL6 controls, as evidenced by similar gross splenic morphology, spleen weights, and total splenocyte numbers (Fig 6A-C). These findings indicate that IL-22 does not substantially influence the overall lymphoproliferative response induced by MHV68 infection.

We next examined the splenic B cell compartment to determine whether the reduction in viral latency observed in IL-22^-/-^ mice (Fig 3) reflected a global defect in B-cell expansion. Compared with mock-infected controls, MHV68 infection induced a robust increase in the absolute numbers of total B220 + B cells. However, no significant differences were observed between infected BL6 and IL-22^-/-^ mice (Fig 6D). These findings indicate that the initial activation and accumulation of splenic B cells, the principal reservoir of latent MHV68 infection, occur independently of IL-22 signaling.

Gammaherpesviruses specifically usurp germinal center B cell differentiation to establish long-term latent infection in memory B cells [56,57]. At the peak of latency (16dpi), the majority of latent viral reservoir is hosted by the germinal center B cells [56,57], with CD4 + T follicular helper cells (Tfh) playing an essential role in the MHV68-driven germinal center response [58,59]. We therefore investigated whether IL-22 contributes to the MHV68-driven germinal center response. In contrast to the comparable total B cell numbers in the spleen (Fig 6D), IL-22^-/-^ mice exhibited a significant reduction in both the frequency and absolute number of germinal center B cells at 16 dpi (Fig 6E-G). Consistent with this phenotype, infected IL-22^-/-^ mice also displayed significantly reduced frequencies and absolute numbers of Tfh cells compared with infected BL6 controls (Fig 6H-J). Together, these findings demonstrate that, IL-22 expression supports the MHV68-driven germinal center response during chronic MHV68 infection.

To determine whether the impaired germinal center response persisted during long-term infection, we examined the splenic B-cell compartment at 42 dpi. By this stage of infection, the pronounced splenomegaly observed at 16 dpi (Fig 6A, B) had largely resolved in both genotypes, with comparable total splenocyte numbers, and B220 ⁺ B-cell counts (Fig 6K, L). Consistent with the contraction of the germinal center response during long-term infection, the frequency and absolute number of germinal center B cells were similar between BL6 and IL-22^-/-^ mice at 42 dpi (Fig 6M-P). These findings suggest that IL-22 primarily contributes to the establishment and early expansion of the virus-driven germinal center response rather than its long-term maintenance.

### IL-22 deficiency is associated with reduced systemic BAFF levels but does not alter the balance between germinal center compartments

**Fig 7:**
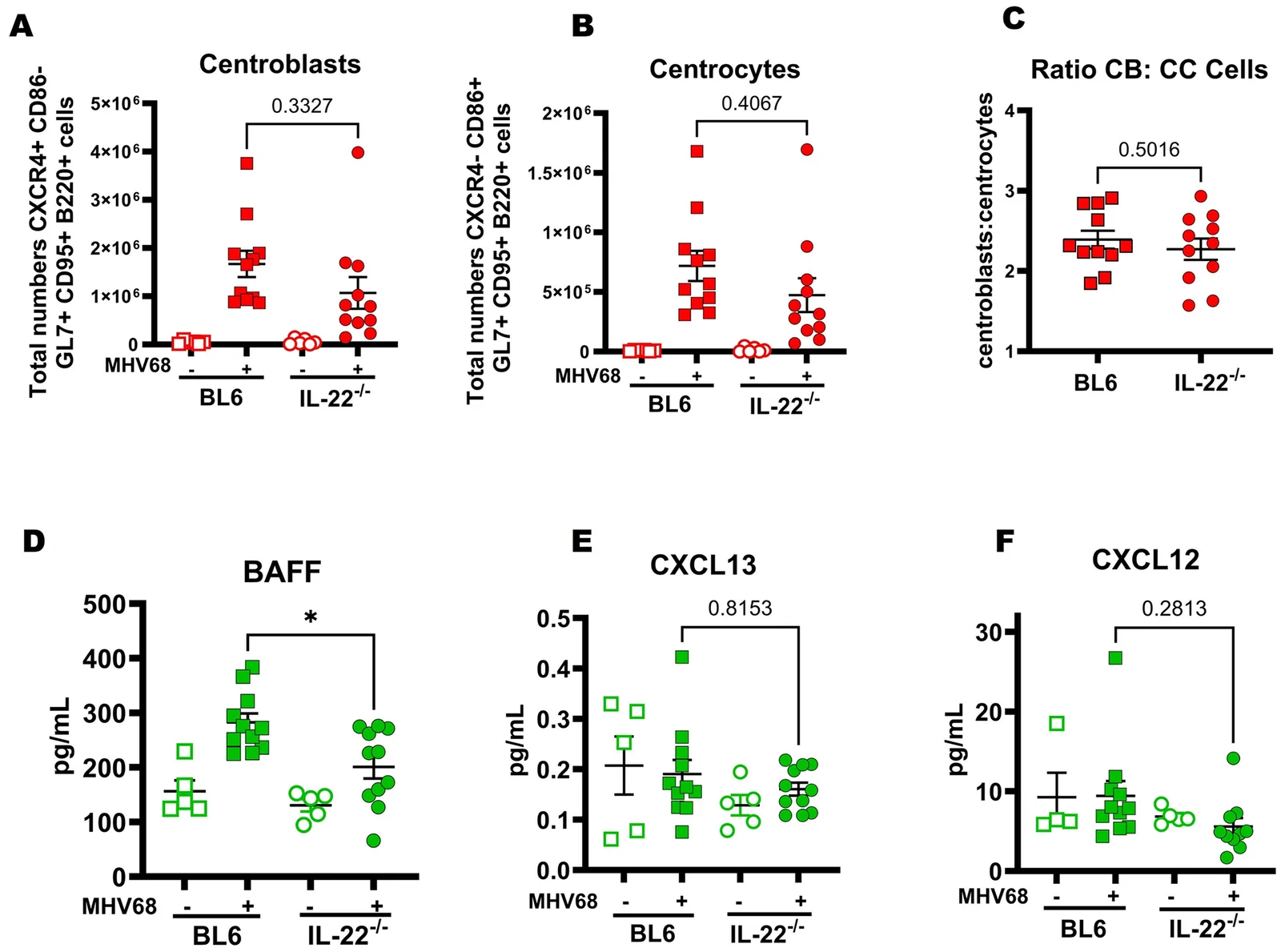
IL-22 deficiency is associated with reduced systemic BAFF levels without altering the balance between germinal center compartments. BL6 and IL-22^-/-^ mice were infected as described in the legend to Fig 1 and analyzed at 16 days post-infection (dpi), corresponding to the peak of latency. **(A, B)** Total numbers of centroblasts (defined as CXCR4 + CD86-) (**A**) and centrocytes (defined as CXCR4-CD86+) (**B**) within the germinal center B-cells (defined as B220 + GL7 + CD95+) compartment were determined by flow cytometry. **(C)** Centroblast-to-centrocyte ratio in infected BL6 and IL-22^-/-^ mice. **(D–F)** Serum concentrations of BAFF **(D)**, CXCL12 **(E)**, and CXCL13 (**F**) were quantified using a flow cytometry-based multiplex bead assay. Each symbol represents an individual mouse. Data are pooled from two to three independent experiments with 2–4 mice per group. Mean and standard error of the mean are shown. *P < 0.05.

To further characterize the germinal center defect observed in IL-22 deficient mice, we quantified the two major germinal center B-cell subsets, centroblasts and centrocytes. Centroblasts are rapidly proliferating germinal center B cells that undergo clonal expansion and somatic hypermutation within the dark zone, whereas centrocytes represent the more differentiated light-zone population that undergoes affinity-based selection and ultimately gives rise to memory B cells and plasma cells [60]. Compared with infected BL6 mice, infected IL-22^-/-^ mice exhibited lower numbers of both centroblasts and centrocytes; however, these differences did not reach statistical significance (Fig 7A, B). To determine whether IL-22 deficiency preferentially affected either stage of GC differentiation, we next calculated the centroblast-to-centrocyte ratio. This ratio was comparable between infected BL6 and IL-22^-/-^ mice (Fig 7C), indicating that IL-22 deficiency does not selectively alter the balance between the dark- and light-zone GC compartments but instead broadly attenuates the germinal center response.

To investigate potential mechanisms underlying this phenotype, we quantified serum levels of BAFF, CXCL12, and CXCL13, cytokines known to regulate B-cell survival, lymphoid organization, and germinal center maintenance [61]. BAFF is a key survival factor that promotes B-cell activation and differentiation, whereas CXCL12 and CXCL13 regulate the migration and positioning of B cells and T follicular helper cells within germinal centers. Notably, MHV68-infected IL-22^-/-^ mice exhibited significantly reduced circulating BAFF levels compared with infected BL6 controls (Fig 7D). In contrast, serum CXCL12 and CXCL13 displayed only modest downward trends that did not reach statistical significance (Fig 7E–F). This observation is consistent with our finding that the proportions of the two principal germinal center compartments, centroblasts and centrocytes, were largely preserved in IL-22-deficient mice (Fig 7A-C), suggesting that IL-22 deficiency does not markedly disrupt germinal center organization. Instead, the selective reduction in BAFF suggests that diminished B-cell survival and differentiation signals may contribute to the impaired germinal center response observed in IL-22-deficient mice.

Collectively, these findings indicate that IL-22 deficiency does not preferentially affect a specific stage of germinal center differentiation but is associated with reduced systemic BAFF levels, supporting a role for IL-22 in promoting the overall expansion of the virus-driven germinal center response during chronic MHV68 infection.

### IL-22 selectively supports activated B-cell subsets during chronic MHV68 infection

**Fig 8:**
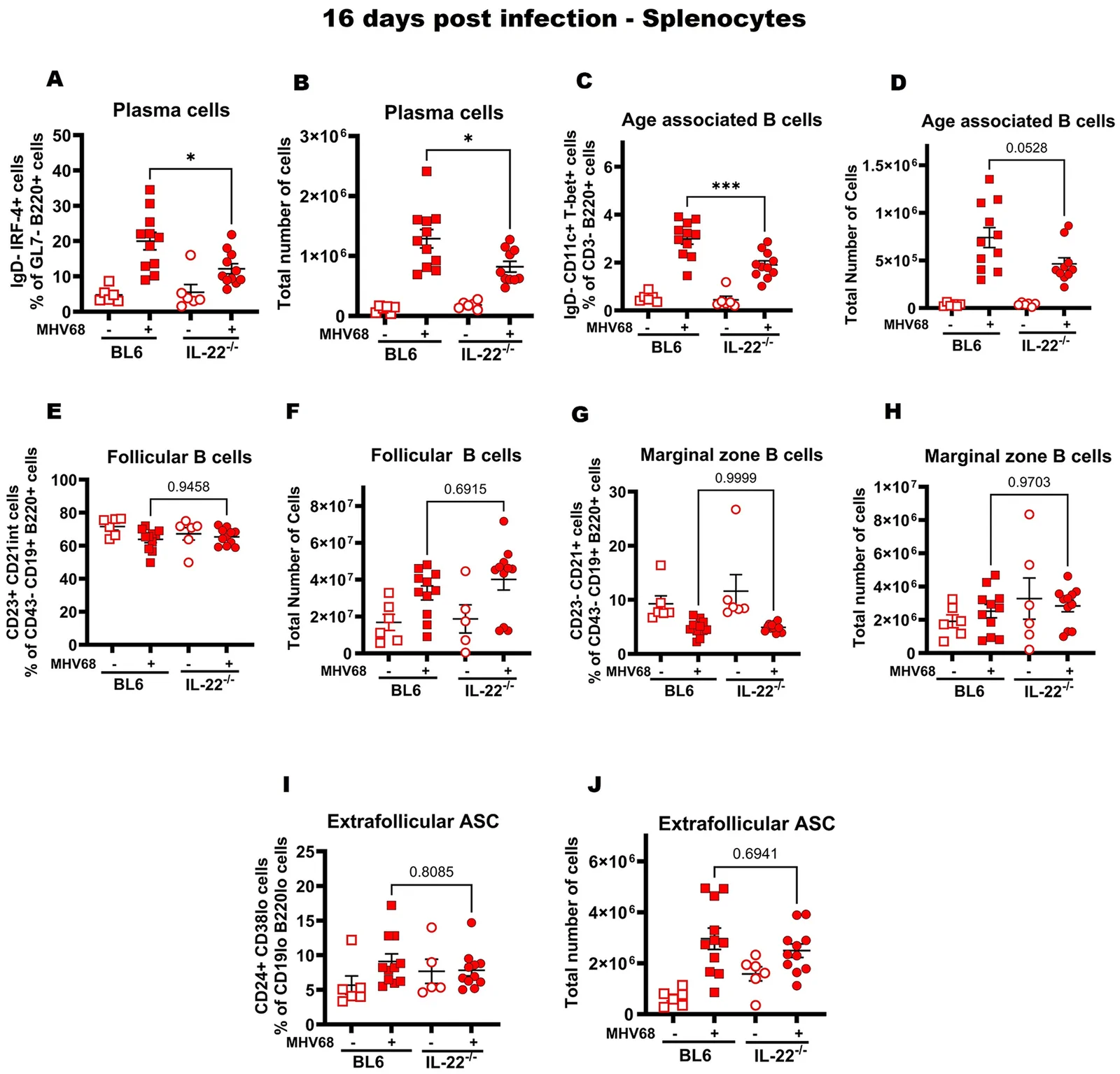
IL-22 deficiency selectively impairs activated B-cell subsets during chronic MHV68 infection. BL6 and IL-22^-/-^ mice were infected as described in Fig 1 and analyzed at 16 days post-infection (dpi), **(A, B)** Frequencies (**A**) and total numbers (**B**) of plasma cells (defined as B220 + GL7-IgD-IRF4 + cells) were determined by flow cytometry. **(C, D)** Frequencies (**C**) and total numbers (**D**) of age-associated B cells (defined as B220 + IgD-CD11c+T-bet+ cells). **(E–H)** Frequencies and total numbers of follicular B cells **(E, F)** (defined as B220 + CD19 + CD43-CD23 + CD21int cells) and marginal zone B cells (defined as B220 + CD19 + CD43-CD23-CD21 + cells) **(G, H)**. **(I, J)** Frequencies (**I**) and total numbers (**J**) of extrafollicular antibody-secreting cells (defined as B220loCD19loCD24 + CD38lo expressing cells). Each symbol represents an individual mouse. Data are pooled from three independent experiments with 2–4 mice per group. Mean and standard error of the mean are shown. *P < 0.05, ***P < 0.001.

To further define the impact of IL-22 deficiency on B-cell differentiation during chronic infection, we quantified additional activated B-cell populations within the spleen. Consistent with the diminished germinal center response observed in IL-22^-/-^ mice, both the frequency and absolute number of plasma cells were significantly reduced compared with infected BL6 controls (Fig 8A,B). Likewise, the frequency of age-associated B cells (ABCs), a population known to expand during chronic viral infection and inflammation, was significantly reduced in IL-22^-/-^ mice (Fig 8C). Although the total number of ABCs also trended lower in infected IL-22^-/-^ mice relative to infected BL6 controls, this difference did not reach statistical significance (Fig 8D).

In contrast, the frequencies and total numbers of follicular (FO) and marginal zone (MZ) B cells were comparable between infected BL6 and IL-22^-/-^ mice (Fig 8E-H), indicating that IL-22 deficiency does not broadly impair maintenance of conventional B-cell subsets. Similarly, the frequencies and total numbers of extrafollicular antibody-secreting cells remained unchanged between genotypes (Fig 8I,J).

Collectively, these findings demonstrate that IL-22 deficiency selectively impacts activated B-cell populations associated with chronic MHV68 infection, including germinal center B cells, plasma cells, and ABCs, while sparing major resting B-cell subsets. These data further suggests that IL-22 contributes to the expansion and maintenance of virus-driven activated B-cell populations during chronic infection.

### IL-22 supports virus-specific and polyclonal humoral responses during chronic MHV68 infection

Having observed a significant reduction in the germinal center B cells, T follicular helper cells, and plasma cells in MHV68-infected IL-22^-/-^ mice (Figs 6–8), we next assessed the functional consequences of these defects on humoral immunity. Gammaherpesvirus infection is characterized by robust B-cell activation that generates both virus-specific antibody responses and extensive polyclonal antibody production directed against irrelevant and self-antigens [62,63].

**Fig 9:**
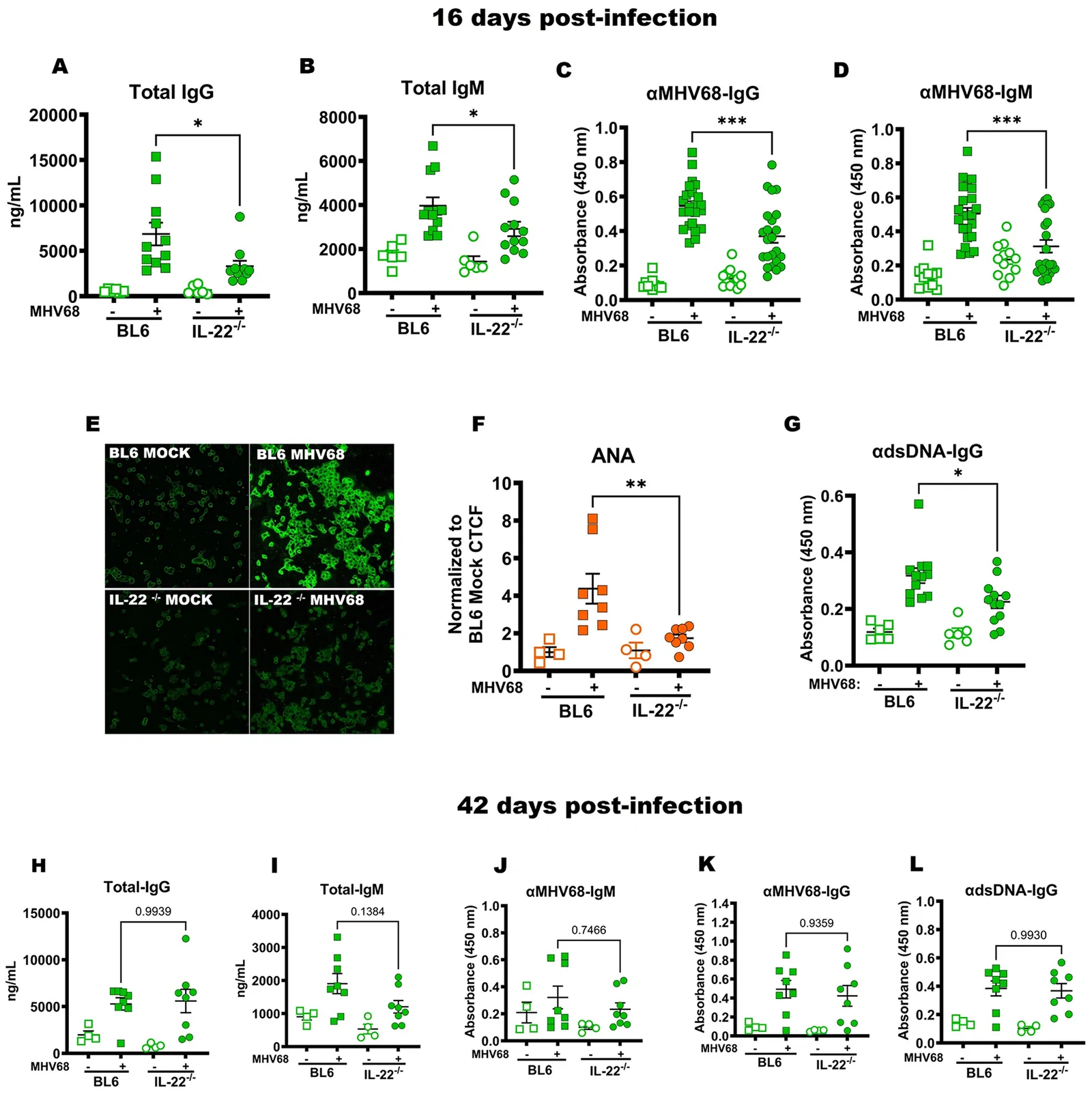
IL-22 deficiency reduces virus specific and polyclonal humoral response during chronic infection. BL6 and IL-22^-/-^ mice were either mock treated or infected as described in the legend to Fig 1. Serum was collected at 16 days post-infection (dpi; peak latency) and 42 dpi (long-term latency) to evaluate humoral immune responses. **(A–D)** Serum concentrations of total IgG **(A)**, total IgM **(B)**, MHV68-specific IgG **(C)**, and MHV68-specific IgM (**D**) at 16 dpi were determined by ELISA. **(E)** Anti-nuclear antibody (ANA) reactivity at 16 dpi was quantified by indirect immunofluorescence using HEp-2 cell substrates. IgG fluorescein isothiocyanate (FITC)-conjugated antibody was used for detection **(F)** ANA staining quantification of the sera from mock- and MHV68-infected BL6 and IL-22^-/-^ mice by corrected total cell fluorescence (CTCF) **(G)** Serum anti-double-stranded DNA (anti-dsDNA) IgG levels at 16 dpi were determined by ELISA. **(H–K)** Serum concentrations of total IgG **(H)**, total IgM **(I)**, MHV68-specific IgG **(J)**, and MHV68-specific IgM (**K**) at 42 dpi were determined by ELISA. **(L)** Serum anti-dsDNA IgG levels at 42 dpi. Data are pooled from two to five independent experiments, with each symbol representing an individual mouse. Mean and standard error of the mean are shown. *P < 0.05, **P < 0.01, ***P < 0.001.

Consistent with the diminished germinal center B and plasma-cell compartments observed in IL-22^-/-^ mice, serum concentrations of total IgG and IgM were significantly reduced in infected IL-22^-/-^ mice compared with infected BL6 controls at 16 dpi (Fig 9A,B). We next examined virus-specific humoral immunity by quantifying MHV68-specific antibody responses. MHV68-specific IgG and IgM titers were significantly reduced in infected IL-22^-/-^ mice compared with infected BL6 controls at 16 dpi (Fig 9C,D). These findings indicate that IL-22 contributes to the generation of effective virus-specific antibody responses during the peak phase of chronic infection.

In addition to inducing antiviral antibodies, gammaherpesviruses are well known to drive vigorous polyclonal B-cell activation, resulting in the production of antibodies directed against self-antigens and unrelated foreign antigens [62,64]. This induction of virus-nonspecific polyclonal antibody response forms the basis for the diagnostic assay, where high levels of antibodies directed against horse red blood cells are indicative of a recent EBV infection in humans [65]. To assess the impact of IL-22 deficiency on this component of the humoral response, sera from infected mice were subjected to anti-nuclear antibody (ANA) analysis. Consistent with the reduction in total immunoglobulin levels, MHV68-infected IL-22^-/-^ mice exhibited significantly diminished overall autoreactivity compared with infected BL6 controls (Fig 9E). Moreover, the qualitative pattern of ANA staining was markedly altered, with sera from infected BL6 mice producing robust pan-cellular staining, whereas sera from IL-22^-/-^ mice displayed substantially reduced ANA staining (Fig 9F).

MHV68 infection induces antibodies with specificity against double-stranded DNA (dsDNA) and have been successfully used to assess irrelevant B cell differentiation during infection [7,8,62,64]. Therefore, to further characterize the irrelevant antibody response, we quantified antibodies directed against double-stranded DNA (anti-dsDNA). Whereas infected BL6 mice mounted a robust anti-dsDNA response, anti-dsDNA titers were significantly reduced in infected IL-22^-/-^ mice (Fig 9G). Collectively, these findings demonstrate that IL-22 supports both virus-specific and polyclonal humoral responses during peak chronic MHV68 infection and promotes the expansion of autoreactive antibody repertoires associated with gammaherpesvirus infection.

To determine whether these humoral defects persisted during long-term infection, we quantified total, virus-specific, and autoreactive antibody responses at 42 dpi. Consistent with the normalization of the germinal center response observed at this stage of infection (Fig 6M,N), total IgG, total IgM, MHV68-specific IgG, and MHV68-specific IgM titers were comparable between infected BL6 and IL-22^-/-^ mice (Fig 9H-K). Similarly, anti-dsDNA antibody levels no longer differed between the two genotypes at 42 dpi (Fig 9L). Together, these findings suggest that IL-22 primarily contributes to the establishment and early expansion of virus-driven humoral responses and autoreactive antibody production rather than their long-term maintenance.

## Discussion

The hallmark of gammaherpesvirus infection is the establishment of a permanent latent reservoir [8]. Using the MHV68 model, we identify IL-22 as a previously unrecognized host factor that promotes the establishment and maintenance of chronic gammaherpesvirus infection. We demonstrate that MHV68 infection induces a robust IL-22 response across multiple anatomical sites and cellular compartments, including conventional T cells, innate lymphoid populations, and NK-lineage cells. Importantly, loss of IL-22 does not impair acute viral replication but results in a sustained reduction in latent viral burden at 16 and 42 days post infection. Further, there was a diminished germinal center B cell and T follicular helper cell response, reduced plasma-cell expansion, and impaired virus-specific and polyclonal antibody production at 16 days post infection. Together, these findings establish IL-22 as a key regulator of the immune microenvironment that supports efficient establishment of chronic gammaherpesvirus infection.

Our findings expand the understanding of IL-22 by identifying a previously unknown proviral role for this cytokine during gammaherpesvirus infection. While IL-22 is traditionally recognized for its host-protective and regenerative functions at mucosal barriers, particularly against bacterial and fungal pathogens, its biological activities are increasingly appreciated to be highly context-dependent [66]. A major contribution of this study is the identification of IL-22 axis as a host pathway that supports chronic gammaherpesviruses infection. Although the dual nature of IL-22 has been extensively explored in inflammatory and infectious settings, its role during gammaherpesvirus infection has not previously been examined. Our findings provide the first evidence that, in the context of gammaherpesvirus infection, IL-22 shifts from predominantly a barrier-protective factor to a facilitator of viral latency.

### Mapping the IL-22 Response during gammaherpesvirus infection

Our study demonstrates that MHV68 infection elicits IL-22 production from diverse array of immune populations, including CD4 + T cells, CD8 + T cells, γδ T cells, NK cells, NKT cells, and ILC3s (Fig 1). The broad distribution of IL-22-producing populations suggests that this cytokine participates in both innate and adaptive phases of the antiviral response. Whether distinct cellular sources dominate distinct stages of infection remains an important question for future investigation.

IL-22 induction is a common feature of host responses to diverse pathogens, ranging from bacterial infections such as *Citrobacter rodentium* [67] *and Klebsiella pneumoniae* [68] to respiratory viral infections including Influenza [69,70]. However, the temporal and spatial distribution of IL22 observed during MHV68 infection is noteworthy. We observed elevated IL-22 levels in the lungs, spleen, and serum during infection, with the most pronounced increases occurring during the chronic phase at 16 days post infection (Fig 1D-R). This pattern suggests that IL-22 may be especially important during the transition from acute infection to the establishment of chronic viral infection.

In many acute mucosal infections, innate lymphoid cells (ILCs), particularly ILC3s, serve as the early producers of IL-22 and promote epithelial barrier integrity [71,72]. Consistent with these observations, we identified ILC3s among the cellular sources of IL-22 during MHV68 infection (Fig 1L, 1N). However, our analyses also revealed substantial contributions from adaptive immune populations, including virus-specific CD4⁺ and CD8 ⁺ T cells (Fig 1O-1R). This persistent IL-22 response creates an intriguing paradox. Immune populations activated to control infection simultaneously generate a cytokine environment that appears to support long-term viral infection. We propose that this represents a form of viral exploitation of a host pathway normally involved in tissue repair, barrier maintenance, and lymphoid organization. Although gammaherpesviruses do not encode a recognizable IL-22 homolog, MHV68 appears to benefit from the host IL-22 response, thereby creating an environment conducive to the establishment of a stable latent reservoir.

### How IL-22 promotes the establishment of chronic gammaherpesvirus infection

A central finding of this study is that IL-22 promotes the establishment of chronic gammaherpesvirus infection. One possible explanation for the reduced latent viral burden observed in IL-22^-/-^ mice was that IL-22 enhances early viral replication, thereby increasing the pool of infected cells available to seed latency. However, analysis of pulmonary viral titers and expression of the lytic viral gene *ORF9* at multiple acute time points demonstrated that acute MHV68 replication proceeds normally in the absence of IL-22 (Fig 2). These findings indicate that IL-22 is dispensable for primary lytic infection and instead exerts its effects during the establishment of chronic infection.

Despite normal acute replication, IL-22-deficient mice exhibited a sustained reduction in latent viral burden within both the spleen and peritoneal cavity during peak latency and long-term infection (Fig 3). Consistent with the reduced frequency of latently infected cells (Fig 3A, C, E, and F), reactivation frequencies were also diminished (Fig 3C, D). Importantly, our data do not necessarily suggest that IL-22 directly regulates the intrinsic capacity of MHV68 to reactivate. Rather, the reduced reactivation frequencies observed in IL-22^-/-^ mice are most likely a consequence of the smaller latent reservoir available to undergo reactivation. Together, these findings support a model in which IL-22 facilitates the efficient establishment of the latent viral reservoir that subsequently persists throughout chronic infection.

### IL-22 promotes chronic infection without broadly impairing antiviral immunity

An important finding of our study is that IL-22 promotes chronic gammaherpesvirus infection without globally altering antiviral immune responses. Despite the reduction in latent viral burden, IL-22-deficient mice displayed normal acute viral replication (Fig 2), preserved activation of the total CD8 ⁺ T-cell compartment (Fig 4A-D), intact IFNγ production by virus-responsive splenic CD8 ⁺ T cells, and no detectable changes in circulating inflammatory cytokines, including IFNγ, TNFα, and IL-6 (Fig 5A-F). Instead, the reduction in virus-specific CD8 ⁺ T cells observed in both the spleen and peritoneal cavity closely paralleled the diminished latent viral burden, suggesting that these changes are more likely a consequence of reduced antigenic stimulation than an intrinsic defect in CD8 ⁺ T-cell differentiation or effector function. Together, these findings indicate that IL-22 is not required for the generation or maintenance of broad antiviral immunity but instead promotes chronic infection by shaping the immunological environment that supports viral latency. These observations, in context of gammaherpesvirus infection, distinguish IL-22 from cytokines that directly regulate antiviral effector responses. This selective role is particularly noteworthy because it suggests that therapeutic targeting of IL-22 may reduce chronic viral latency without substantially compromising systemic antiviral immunity.

### IL-22 supports germinal center biology

The most prominent immunological phenotype observed in IL-22-deficient mice was a selective disruption of the virus-driven germinal center response. While splenomegaly, spleen weights, total splenocyte expansion, and overall B-cell accumulation remained intact during peak latency (Fig 6A-D), IL-22 deficiency resulted in reduced germinal center B cells (Fig 6E-G), diminished T follicular helper cells (Fig 6H-J), decreased plasma-cell expansion (Fig 8A, B), and reduced accumulation of age-associated B cells (Fig 8C, D). Notably, the defect in germinal center response was most pronounced during peak latency and largely resolved during long-term infection, suggesting that IL-22 primarily contributes to the establishment and early expansion of the germinal center response rather than its long-term maintenance. Given that germinal center B cells represent the major reservoir of latent MHV68 during chronic infection [55,56], the diminished latency observed in IL-22^-/-^ mice are likely linked to the impaired expansion of this critical cellular niche.

Although the precise cellular targets of IL-22 remain unresolved, our findings provide several mechanistic clues. Because hematopoietic cells generally lack functional IL-22Rα expression [73], the observed phenotypes are unlikely to result from direct signaling within B- or T-cell compartments. Instead, IL-22 likely acts on non-hematopoietic populations that help shape the germinal center microenvironment. Consistent with this possibility, infected IL-22-deficient mice exhibited significantly reduced systemic BAFF levels (Fig 7D). BAFF is a critical regulator of B-cell survival, differentiation, and germinal center maintenance [74], suggesting that reduced BAFF availability may contribute to the impaired germinal center response observed in the absence of IL-22. Although these findings do not establish a direct mechanistic pathway, they identify the IL-22–BAFF axis as a potential link between IL-22 signaling and the establishment of chronic gammaherpesvirus infection. Future studies employing cell-specific deletion of IL-22Rα will be necessary to identify the relevant IL-22-responsive populations and define the molecular pathways that connect IL-22 signaling to germinal center biology.

### IL-22 supports virus-specific and polyclonal humoral responses during chronic infection

Gammaherpesviruses are unique in their ability to induce extensive B-cell activation and robust germinal center responses that support both establishment of chronic infection and antibody production [62,75]. Consistent with the diminished germinal center and plasma-cell responses observed in IL-22-deficient mice (Figs 6 and 8), we identified substantial defects in humoral immunity during peak chronic infection. In addition to reduced total IgG and IgM concentrations (Fig 9A and 9b), IL-22^-/-^ mice exhibited significantly reduced MHV68-specific IgG and IgM responses (Fig 9C and 9D). These findings indicate that IL-22 supports not only to generalized B-cell activation but also to the generation of effective virus-specific humoral immunity during chronic infection.

In parallel with the reduction in antiviral antibody responses, IL-22 deficiency markedly attenuated the autoreactive antibody response typically associated with gammaherpesvirus infection (Fig 9E-G). Both overall anti-nuclear antibody reactivity (Fig 9E and 9F) and anti-dsDNA antibody (Fig 9G) levels were significantly reduced during peak latency at 16 days post infection. These observations are consistent with previous studies demonstrating that gammaherpesviruses drive a transient breakdown of B-cell tolerance through exaggerated germinal center activity and polyclonal B-cell activation [62,75,76] Our findings therefore suggest that IL-22 broadly supports the expansion of both antigen-specific and autoreactive B-cell populations generated during chronic infection.

Gammaherpesviruses are unique in their ability to induce a robust germinal center response, which supports a majority of the latent viral reservoir [56,62,64]. These viruses also drive an increase in polyclonal antibodies directed against non-relevant foreign antigens and even self-directed antigens [17,64]. Our findings identify IL-22 as an important component of this process. By supporting germinal center expansion, plasma-cell differentiation, humoral immunity, and latent reservoir establishment, IL-22 contributes to an immune environment that favors chronic viral infection. While the precise cellular targets of IL-22 signaling remain to be identified, our data support a model in which IL-22 acts indirectly through the lymphoid microenvironment to promote virus-driven B-cell differentiation and chronic infection.

Finally, the reduction in in latent viral burden, germinal center responses, and autoreactive antibody production observed in infected IL-22^-/-^ mice raises the possibility that IL-22 axis may represent a therapeutic target for limiting gammaherpesvirus-associated immunopathology. Importantly, disruption of IL-22 signaling reduced chronic infection without impairing acute viral control, suggesting that IL-22 primarily influences the establishment of chronic viral infection rather than fundamental antiviral defense mechanisms. Although further studies will be required to determine whether similar pathways operate during human gammaherpesvirus infections such as EBV and KSHV, our findings provide a framework for investigating whether modulation of IL-22 signaling can reduce chronic viral infection and associated dysregulated humoral responses while preserving protective immunity. Understanding these mechanisms may ultimately facilitate the development of therapeutic strategies aimed at limiting the long-term consequences of chronic gammaherpesvirus infection.

## Materials and methods

### Ethics statement

All experimental manipulations of mice were approved by the Institutional Animal Care and Use Committee of Western Michigan University Homer Stryker M.D. School of Medicine (2022-0026).

### Animals

C57BL/6J and IL-22^-/-^ (C57BL/6-*Il22*^*tm1.1(icre)Stck*^/J, stock # 027524) mice were ordered from The Jackson Laboratories (Bar Harbor, ME). All mice were housed and bred in a specific-pathogen-free facility. Both male and female mice were used with no gender-specific phenotypes noted.

### Infections

MHV68 viral stock was prepared, and the virus titers on NIH 3T12 cells were determined. Six to ten-week-old mice were subjected to light anesthesia and intranasally inoculated with 1,000 PFU of MHV68 diluted in 15 µl sterile serum-free Dulbecco’s modified Eagle’s medium (DMEM). The spleen, lung, and peritoneal cells were harvested from euthanized mock-treated and MHV68-infected animals at indicated times postinfection. Mice were humanely euthanized using CO2 inhalation. Mice were bled before euthanasia via submandibular vein sampling, and the serum was collected using BD Microtainer blood collection tubes (Becton, Dickinson and Company, Franklin Lakes, NJ).

### Plaque assay for acute viral replication

Lungs were harvested from MHV68-infected BL6 and IL-22^-/-^ mice at 7 and 9 days post-infection and homogenized in sterile serum-free DMEM. Tissue homogenates were clarified by centrifugation, and supernatants were serially diluted (10 fold) before plating onto confluent NIH 3T12 fibroblast monolayers. After adsorption, cells were overlaid with methylcellulose-containing medium and incubated until plaques became visible. Monolayers were then fixed and stained with phenol red, and plaques were enumerated to determine infectious viral titers. Viral titers were calculated as plaque-forming units (PFU) per lung.

### Reverse transcription-quantitative PCR

Total RNA was isolated from the lungs of infected BL6 and IL-22^-/-^ mice at 7 and 9 dpi mice using TRIzol according to the manufacturer’s instructions (Invitrogen, Carlsbad, CA). RNA (2 µg) was treated with RNase-free DNase I (ThermoScientific-Waltham, MA) and split into two reaction mixtures. The first half was subjected to reverse transcription using RevertAid First Strand cDNA Synthesis Kit (ThermoScientific, Waltham, MA) and the second half of the DNase-treated RNA was subjected to mock reverse transcription in the absence of the enzyme (-RT control). cDNA, including -RT controls, was diluted 2-fold and measured, in duplicate for each dilution, by real-time PCR using the AZURE CIELO real-time PCR detection system (Azure Biosystems-Dublin, CA). The relative abundance of ORF9 was normalized to corresponding GAPDH levels. The comparative threshold cycle (ΔΔCT) method was used to quantify relative abundance of each cDNA. Primers used for q-PCR were as follows: ORF9 Forward: 5′- TGCATGCAAGTTTGTCCAGTCT-3’; ORF9 Reverse: 5′ CTTCCCCCAGTTACTCATTGTTTG-3’; GAPDH Forward: 5′- ACCACAGTCCATGCCATCAC-3’; GAPDH Reverse: 5′- TCCACCACCCTGTTGCTGTA-3’.

### Limiting dilution assays

The frequency of virally infected cells (cells harboring viral DNA) was determined by limiting- dilution (LD) PCR analysis, while the frequency of ex vivo reactivation to identify cells capable of producing infectious virus was determined by limiting-dilution-assay. Spleens from MHV68 infected C57BL/6 and IL-22^-/-^ mice were mashed through 100-micron cell strainer (Fischer). Red blood cells were lysed by incubating in 1–2 ml of RBC lysis buffer (Sigma) for 3 minutes at 37°C. RBC lysis was neutralized by adding 18 mL DMEM and followed by 10-minute centrifugation at 1000rpm. The supernatant was discarded and splenocytes were resuspended in 12–16 ml of serum supplemented DMEM depending upon the pellet size. Splenocytes were then filtered through 100-micron strainer (Fischer) to reduce clumping. Peritoneal lavage cells were isolated in 10ml of DMEM. The cells were centrifuged at 100rpm for 10 minutes. The supernatant was discarded and cells re-suspended in 0.5- 3ml of serum supplemented DMEM depending upon the pellet size. Limiting-dilution (LD)-PCR analysis were performed as previously described [77]. Briefly, splenocytes and peritoneal cells were pooled from all infected mice in each experimental group (three to five mice/group) and six threefold serial dilutions of latently infected cells were carried out in the background of uninfected 3T12 fibroblasts. Twelve replicates per dilution were loaded in 96 well PCR plate. The cells were digested overnight in presence of proteinase K and then subjected nested PCR using specific primers against the viral genome. Single-copy sensitivity and the absence of false-positive amplicons were confirmed using control standards. The samples were run on ethidium bromide stained 2% agarose gel for quantitation.

Ex-vivo reactivation efficiency was determined as previously described [77]. Briefly, eight 2-fold serial dilutions of splenocytes or peritoneal cells from infected mice were plated on mouse embryonic fibroblasts (MEF) monolayers at 24 replicates per dilution. The presence of preformed infectious virus was detected by plating four 2-fold serial dilutions of mechanically disrupted splenocytes or peritoneal cells on MEF monolayers in parallel. Viral reactivation as indicated by the cytopathic clearing of MEFs was assessed on day 21 of culture. All replicates were scored in a binary fashion for the presence of live fibroblasts (no viral reactivation/replication) or their absence (cytopathic effect driven by lytic replication).

### Flow cytometry

Single-cell suspensions of splenocytes and peritoneal cells from individual mice were prepared in fluorescence-activated cell sorting (FACS) buffer (phosphate-buffered saline, 2% fetal bovine serum, 0.05% sodium azide). 2x10^6^ cells were treated with Fc block (24G2) before extracellular staining for 30 min on ice. Data were acquired using an Attune NxT flowcytometer (Thermo Fisher Scientific, Waltham, MA) and analyzed using FlowJo software (Becton, Dickinson & Company, Ashland, OR). Following antibodies used in the study were purchased from BioLegend (San Diego, CA): CD3 (17A2), CD4 (RM4–5), CD5 (53-7.3), CD8a (53-7.3), CD11b (M1/70), CD11c (N418), CD19 (6D5), CD21 (7E9), CD23 (B3B4), CD24 (M1/69), CD38 (90), CD43 (S11), CD44 (IM7), CD45 (30-F11), CD49b (DX5), CD86 (GL-1), CD127 (A7R34), CXCR4 (L276F12), CXCR5 (L138D7), PD-1 (29f.1A12), B220 (RA3-6B2), GL7 (GL-7), CD44 (IM7), IL-22 (IL22JOP), RORγT (Q31-378), TCRβ (H57-597), TCRγδ (GL3), Thy1 (53-2.1), IFN-g (XMG1.2), IRF-4 (IRF4.3E4), T-bet (4B10), IgD (11-26c.2a), and Lineage (CD3-17A2, Ly6C/Ly6G-RB6-8C5, CD11b-M1/70, B220-RA3-6B2,and Ter-119). CD95 (JO2) was purchased from BD Horizon (San Jose, CA). MHV68 Orf6 tetramer was obtained through the NIH Tetramer core (Atlanta, GA). Compensation controls were done using OneComp eBeads (Thermo Fisher Scientific, Waltham, MA). Briefly, negative control (beads alone) were used to establish a baseline PMT (photomultiplier tube) voltage and fluorescent background. Positive controls for each fluorochrome (beads with a single fluorochrome) were used to establish spill-over of the individual fluorochrome into the other channels being used. PMT values are adjusted for each fluorochrome to minimize spillover.

### T-cell phenotyping

For T-cell phenotyping, single-cell suspensions of splenocytes and peritoneal cells were plated at 4x10^6^ nucleated cells/mL in a 96-well plate. Non-specific stimulation of the cells was carried out using 10 ng/mL of phorbol 12-myristate 13-acetate (PMA; P8139-5MG; Sigma-Aldrich, St. Louis, MO), 1 mg/mL of ionomycin (I0634-1MG; Sigma-Aldrich), and 10 mg/mL of brefeldin A (420601; BioLegend, San Diego, CA) in DMEM with 10% FBS for 4 h at 37°C. For virus-specific CD4 + T-cell stimulation, 2.5 mg/mL of MHV68-specific viral peptide GP150 [78] (GenScript, Piscataway, NJ), and for virus-specific CD8 + T-cell stimulation 10mg/mL of ORF6 and ORF61 viral peptides (Thermo Fisher Scientific, Waltham, MA) were used in conjugation with 10mg/mL of brefeldin A (420601; BioLegend) in DMEM with 10% FBS for 6 h at 37°C. Following re-stimulation, cells were washed in FACS buffer before being treated with Fc block (24G2) and subjected to extracellular staining with an optimal antibody concentration for 30 min on ice. After extracellular staining, the cells were fixed and permeabilized using the BD Cytofix/Cytoperm kit (554714; Fisher Scientific, Hampton, NH). The cells were then intracellularly stained with an optimal antibody concentration for 30 min on ice. Data were acquired using Attune flow cytometer (Thermo Fisher) and analyzed using FlowJo software (Becton, Dickinson & Company, Ashland, OR).

### Enzyme-linked immunosobent assay (ELISA)

Total, MHV68-specific, and dsDNA immunoglobulin levels were determined as previously described [79]. Briefly, Nunc Maxisorp plates (Fisher Scientific, Pittsburg, PA) were coated with either anti-IgG (heavy and light) or anti-IgM antibodies (Jackson ImmunoResearch, West Grove, PA), UV-irradiated MHV68 virus stock in PBS (740,000 micro joules/cm^2^X2) (Stratalinker UV Crosslinker 1800; Agilent Technologies, Santa Clara, CA), or dsDNA from Escherichia coli (12.5mg/ml; Sigma-Aldrich, St. Louis, MO) overnight at 4°C. Plates were washed with PBS-Tween (0.05%) and blocked for 1h with PBS-Tween (0.05%)-BSA (3%). This was followed by incubation with fivefold serial dilutions of serum in PBS-Tween (0.05%)-BSA (1.5%) for 2h and then washing with PBS-Tween (0.05%). Horseradish peroxidase (HRP)-conjugated goat anti-mouse total IgG (heavy and light chain [H1L]) or IgM (Jackson ImmunoResearch, West Grove, PA) along with 3,39,5,59-tetramethylbenzidine substrate (Life Technologies, Gaithersburg, MD) was used to detect the bound antibody. HRP enzymatic activity was stopped by the addition of 1 N HCl (Sigma-Aldrich, St. Louis, MO), and the absorbance was read at 450nm on BioTek EPOCH2 Plate Reader (Agilent Technologies, Santa Clara, CA).

### Multi-analyte flow assay

Quantification of soluble IL-22, TNFα, IFNγ, and IL-6 concentrations in the serum were measured via LEGENDplex Mouse Th17 Panel (7-plex) multi-analyte flow assay kit (Cat# 741048) BioLegend (San Diego, CA) and that of BAFF, CXCL12 and CXCL13 were measured using a custom ordered LEGENDplex flow assay kit as no kit at the time contained all three of those analytes together. Assays were performed according to manufacturer directions and analyzed on AURORA spectral cytometer (Cytek, San Diego, CA). Data was processed with the LEGENDplex online data analysis software to determine the concentrations of the samples

### ANA panels

Antinuclear antibodies (ANAs) were assessed with an (ANA) test kit (Antibodies Inc., Davis, CA). Following the manufacturer’s protocol, serum was diluted (1:40 in PBS) and incubated over slides coated with fixed HEp-2 cells. Following serum incubation, the slides were rinsed and stained with anti-mouse IgG labeled with Alexa Fluor 488 (H + L) (ThermoScientific, Waltham, MA). Fluorescent images were captured using NIS Elements software. Corrected fluorescence was quantified using ImageJ software from a randomly chosen field of ~ 20 cells in each sample.

### Protein extraction from tissues

For IL-22 detection from tissues, lung and spleen were harvested at the indicated time points post-infection. The tissues were disrupted in NP-40 lysis buffer (Thermo Scientific) and protease inhibitor cocktail (Thermo Scientific) by bead-beating using 1-mm zirconia/silica beads (Biospec Products, Bartelsville, OK). Homogenates were first incubated on ice for 20 minutes to allow complete lysis, followed by centrifugation at 20,000 × g for 20 min. The supernatants were collected, and protein concentrations measured with a protein assay kit (Thermo Scientific). Equal amounts of proteins (100µg) were loaded for IL-22 detection using IL-22 enzyme-limited immunosorbent assay (ELISA) Max Deluxe set (BioLegend, San Diego, CA) according to the manufacturer’s instructions, and Nunc Maxi Sorp flat-bottomed plates (Thermo Fisher Scientific, Waltham, MA).

### Statistical analyses

Statistical analyses were performed using Student’s *t* test when comparing two groups and one-way ANOVA with Tukey’s post hoc test when comparing more than two groups (Prism, GraphPad Software, Inc.).
