# Supplementary material for: IL-22 promotes the establishment of chronic gammaherpesvirus infection by supporting the germinal center response
Source: PLoS Pathog. 2026 Sep 8;22(9):e1014016. doi: 10.1371/journal.ppat.1014016 (PMC13585334; doi:10.1371/journal.ppat.1014016)
Supplement: Supplementary file 1 [file ppat.1014016.s001.pdf]

Dear Editor,

We sincerely thank you and the reviewers for the careful evaluation of our manuscript entitled "IL-22 promotes the establishment of chronic gammaherpesvirus infection by supporting germinal center responses." We greatly appreciate the insightful and constructive comments, which have substantially improved the quality and clarity of our work.

In response to the reviewers' suggestions, we have extensively revised the manuscript and performed numerous additional experiments to address the major concerns. These new studies include analyses of acute viral replication, expanded characterization of IL-22-producing immune cell populations, additional evaluation of virus-specific $CD8^+$ T-cell responses, assessment of $CD8^+$ T-cell effector function and systemic inflammatory cytokines, mechanistic analyses of BAFF, CXCL12, and CXCL13, expanded characterization of activated B-cell subsets, additional analyses of virus-specific and autoreactive antibody responses, and long-term (42 dpi) studies of viral latency, germinal center responses, and humoral immunity. We have also substantially revised the Introduction, Results, Discussion, Abstract, Author Summary, figures, and figure legends to improve clarity and to better align our conclusions with the data.

Below, we provide a detailed point-by-point response to each reviewer comment. All changes have been incorporated into the revised manuscript.

Reviewer 1

Summary

This manuscript investigates the role of IL-22 in murine gammaherpesvirus 68 (MHV68) infection, demonstrating that IL-22 promotes viral latency, germinal center expansion, and polyclonal antibody production. Using IL-22 knockout mice, the authors report reduced latent viral load, impaired reactivation, diminished germinal center responses, and reduced autoantibody production. The study proposes that IL-22 acts as a host factor exploited by gammaherpesviruses to support chronic infection.

The work addresses an interesting question regarding host cytokines that support viral latency, which is relevant to EBV/KSHV biology. In particular, the role of IL-22 in gammaherpesvirus latency has not been extensively explored and represents a potentially novel host pathway influencing viral persistence. The authors examine IL-22 induction after infection, latency frequency, viral reactivation, germinal center responses, CD8 T cell responses and antibody

production. These works provide a broad view of how IL-22 deficiency alters infection. The reduced germinal center response correlates well with reduced latency and reduced polyclonal antibody response, which support the overall model. However, while the study is solid at the descriptive level, the mechanistic depth is limited, and several conclusions are not sufficiently supported by the data.
Overall, the manuscript is potentially suitable for PLOS Pathogens, but major revisions are required to strengthen mechanistic insight and address several conceptual issues.

Response: We sincerely thank the reviewer for the thorough, thoughtful, and constructive evaluation of our manuscript. We greatly appreciate the reviewer's recognition of the novelty and potential significance of identifying IL-22 as a host factor that promotes chronic gammaherpesvirus infection, as well as the positive assessment of the breadth of our analyses examining viral latency, germinal center responses, $CD8^+$ T-cell responses, and humoral immunity.

We also appreciate the reviewer's insightful suggestions regarding the mechanistic basis of IL-22 function and the interpretation of several aspects of the study. In response, we have substantially strengthened the manuscript by performing numerous additional experiments, including analyses of acute viral replication, expanded characterization of IL-22-producing immune cell populations, additional evaluation of virus-specific $CD8^+$ T-cell responses and effector function, mechanistic analyses of BAFF, CXCL12, and CXCL13, expanded characterization of activated B-cell subsets, and additional analyses of virus-specific and autoreactive antibody responses. We have also extensively revised the Abstract, Introduction, Results, Discussion, figures, and figure legends to improve clarity, moderate our conclusions where appropriate, and better define the mechanistic framework of the study.

We believe these extensive revisions have substantially strengthened the manuscript and have addressed the reviewer's major concerns. Our detailed point-by-point responses are provided below.

Major Comment 1

The central conclusion is that IL-22 promotes viral latency by supporting germinal center expansion. However, the study does not determine which cells respond to IL-22? how IL-22 signaling promotes germinal center formation? what downstream pathways are involved?

The manuscript itself notes that B cells lack IL-22Rα, implying the effect must be indirect. But no experiments were done to address stromal cells, follicular dendritic cells, epithelial cells, and cytokine/chemokine changes. Thus, the mechanistic explanation remains speculative.

Response: We thank the reviewer for this thoughtful comment and agree that identifying the IL-22-responsive cellular compartment represents an important next step toward defining the mechanism by which IL-22 promotes chronic gammaherpesvirus infection. Although comprehensive identification of the IL-22-responsive cell population using conditional IL-22Rα-deficient mice or stromal cell-specific approaches is beyond the scope of the current study, we have substantially strengthened the mechanistic framework of the manuscript through several new experiments and an expanded discussion.

First, because IL-22 primarily signals through IL-22Rα expressed on non-hematopoietic cells, we have revised the Discussion to more clearly acknowledge that the effects of IL-22 on germinal center responses are likely indirect and mediated through the lymphoid microenvironment rather than direct signaling to B cells.

Second, to begin exploring mechanisms that could underlie the impaired germinal center response, we performed additional analyses of circulating BAFF, CXCL12, and CXCL13, key regulators of B-cell survival, lymphoid organization, and germinal center maintenance. These studies revealed significantly reduced systemic BAFF levels in MHV68-infected IL-22-deficient mice (new Fig. 7). These findings identify reduced BAFF availability as a potential mechanism contributing to the diminished germinal center response observed in IL-22-deficient mice.

Finally, we have substantially expanded the Discussion to emphasize the indirect nature of IL-22 signaling, discuss potential roles for stromal and other IL-22-responsive non-hematopoietic cells in shaping the germinal center microenvironment, and explicitly acknowledge that defining the relevant IL-22-responsive cellular targets will require future studies using cell type-specific genetic models.

Accordingly, we have moderated our mechanistic conclusions throughout the manuscript and now state that our data identify a potential mechanism linking IL-22 signaling with germinal center expansion rather than establishing a direct causal pathway.

Major Comment 2

The authors interpret IL-22 as a proviral factor promoting latency, but an alternative explanation is not excluded. Specifically, IL-22 may enhance early viral replication, which then secondarily increases the latent reservoir. The manuscript does not measure acute viral titers, early infection levels and lytic replication. These data are necessary before concluding IL-22 specifically promotes latency.

Response: We thank the reviewer for this important suggestion and agree that it was essential to determine whether the reduced latent viral burden observed in IL-22-deficient mice resulted from impaired acute viral replication rather than a direct effect on the establishment of chronic infection. To address this concern, we performed new experiments examining acute MHV68 replication in the lungs, the primary site of lytic infection following intranasal inoculation.

Specifically, we quantified infectious viral titers in the lungs of infected BL6 and IL-22$^{-/-}$ mice at both 7, and, 9 days post-infection (dpi), representing the peak of acute lytic replication and the onset of viral clearance, respectively (new Fig. 2A-B). In addition, we measured ORF9, a viral lytic/ early gene, levels by quantitative PCR at these time points (new Fig. 2C-D). Importantly, neither infectious viral titers nor ORF9 levels differed significantly between BL6 and IL-22$^{-/-}$ mice at either time point, demonstrating that IL-22 deficiency does not impair acute MHV68 replication.

These new findings demonstrate that the reduction in latent viral burden observed at 16 and 42 dpi cannot be attributed to defects in early viral replication. We have therefore revised the Results and Discussion to clearly distinguish the effects of IL-22 on acute lytic infection from its role in promoting the establishment of chronic infection. Accordingly, our conclusions are now based on the observation that IL-22 deficiency selectively reduces chronic viral latency despite normal acute viral replication.

Major Comment 3

The observed germinal center defect may simply reflect reduced antigen load. The authors' interpretation is that IL-22 affects germinal center expansion, which then affect latency. However, the reverse is equally plausible: Reduced viral burden could lead to reduced antigen,

which could reduce germinal centers. Importantly, the CD8 T cell data already suggest reduced antigen load in IL-22 knockout mice. Thus, the causal direction remains unclear.

Response: We thank the reviewer for raising this important point. We agree that distinguishing whether the diminished germinal center (GC) response is the cause or the consequence of the reduced latent viral burden represents a key mechanistic question.

To address this concern, we first performed additional experiments examining acute MHV68 replication. As described above (new Fig. 2), IL-22 deficiency did not alter acute viral titers or viral genome levels at 7 or 9 dpi, indicating that the reduced latent reservoir is not secondary to impaired early lytic replication. Thus, our data support a role for IL-22 during the establishment of chronic infection rather than during the acute phase of infection.

We also expanded our characterization of the chronic immune response. In addition to reduced germinal center B cells and T follicular helper cells, IL-22-deficient mice exhibited reduced plasma-cell expansion (Fig. 8A, B), diminished age-associated B-cell responses (Fig. 8C, D), significantly lower systemic BAFF levels (Fig.7D), and impaired virus-specific and polyclonal antibody responses (Fig. 9A, D). Together, these findings consistently identify defects in multiple components of the germinal center and humoral immune response that accompany the reduced latent reservoir.

Importantly, we agree with the reviewer that the present study does not definitively establish the temporal sequence between reduced latency and impaired germinal center responses. Accordingly, we have moderated our interpretation throughout the manuscript and revised the Discussion to acknowledge that these processes are likely closely interconnected. Rather than concluding that IL-22 directly regulates germinal center expansion, we now state that IL-22 supports germinal center responses associated with the establishment of chronic gammaherpesvirus infection and discuss that future studies using temporally controlled or cell type-specific genetic approaches will be required to determine the precise causal relationship.

Major comment 4

The manuscript states that virus-specific CD4 and CD8 T cells produce IL-22. However, IL-22 can also originate from ILC3 cells, γδ T cells and Th17 cells. A clearer characterization of the cellular sources would strengthen the study.

Response: We thank the reviewer for this valuable suggestion and agree that a more comprehensive characterization of the cellular sources of IL-22 substantially strengthens the

study. To address this comment, we performed additional flow cytometric analyses to identify IL-22-producing immune cell populations during chronic MHV68 infection.

We quantified IL-22-producing γδ T cells, NK cells, NKT cells, and group 3 innate lymphoid cells (ILC3s) in both the spleen and peritoneal cavity at 16 days post-infection. In addition, we expanded our analysis of adaptive immune cells by quantifying virus-specific IL-22-producing $CD4^+$ and $CD8^+$ T cells following ex vivo peptide restimulation. These new data demonstrate that MHV68 infection induces IL-22 production from multiple innate and adaptive immune cell populations across both anatomical sites (new Fig. 1G–R).

Accordingly, the Results, Discussion, Figure 1, Figure 1 legend, and Materials and Methods sections have been extensively revised to incorporate these new findings. We believe these additional experiments provide a much more comprehensive characterization of the cellular sources of IL-22 during chronic MHV68 infection and substantially strengthen the manuscript.

Major comment 5

The manuscript frequently references EBV and KSHV, but all data are from MHV68 infection in mice. There is no evidence that IL-22 affects EBV latency and IL-22 affects KSHV infection. Some supporting evidence from human datasets or literature would strengthen the translational significance.

Response: We thank the reviewer for this important comment and agree that our findings are derived entirely from the MHV68 mouse model and should not be interpreted as direct evidence for a role of IL-22 during human EBV or KSHV infection.

In response to this concern, we have carefully revised the manuscript to moderate the translational interpretation throughout the Introduction, Discussion, Abstract, and Author Summary. We now consistently present MHV68 as a well-established and biologically relevant model for investigating fundamental mechanisms of gammaherpesvirus infection while clearly acknowledging that whether similar IL-22-dependent pathways operate during EBV or KSHV infection remains to be determined.

In addition, we have expanded the Discussion to place our findings within the context of the existing literature on IL-22 biology and gammaherpesvirus infection. We discuss published studies demonstrating elevated circulating IL-22 levels during EBV-associated infectious mononucleosis while emphasizing that direct evidence linking IL-22 to EBV or KSHV latency is

currently lacking. Accordingly, we now state that future studies will be required to determine whether the mechanism identified here is conserved across human gammaherpesviruses.

We believe these revisions more appropriately position the translational significance of our findings while avoiding overinterpretation beyond the scope of the current study.

Minor comment 1

The manuscript contains several redundant statements. The Discussion could be significantly condensed.

Response: We thank the reviewer for this helpful suggestion. In response, we have substantially revised and condensed the Discussion to improve clarity and eliminate redundant statements. The Discussion has been reorganized into focused thematic sections that more clearly highlight the major findings of the study, integrate the newly generated data, and provide a more balanced discussion of the proposed mechanisms and study limitations. We believe these revisions have improved the overall readability and flow of the manuscript while maintaining the scientific context and significance of our findings.

Minor comment 2

Some figures report trends without clear statistical support (e.g., IL-22 levels in spleen at early time points). Statistical methods should be more clearly described.

Response: We thank the reviewer for this important observation. In response, we carefully revised the manuscript to ensure that trends not supported by statistical significance are described appropriately throughout the Results and Discussion. For example, observations such as the increase in splenic IL-22 levels at the early stage of infection are now described as trends that did not reach statistical significance, rather than implying a significant biological effect. We believe these revisions improve the clarity and accuracy of the data presentation.

Minor comment 2

The manuscript states that data hosting is still being determined. PLOS requires data availability upon publication.

Response: We agree with the reviewer's observation. Accordingly, we have uploaded all the data in the revised submission.

Minor comment 3

The authors state “first evidence that IL-22 plays a proviral role in gammaherpesvirus infection”. This should be toned down slightly. While the specific context may be new, IL-22 has already been implicated in viral pathogenesis in other infections.

Response: We thank the reviewer for this helpful suggestion and agree that our original wording was too broad. While IL-22 has previously been implicated in the pathogenesis of several viral infections, its role during gammaherpesvirus infection has not been investigated.

Accordingly, we have revised the manuscript throughout to moderate our claims. Rather than broadly stating that our study provides the "first evidence" of a proviral role for IL-22, we now describe IL-22 as a **previously unrecognized host factor** that promotes chronic gammaherpesvirus infection. In addition, we have expanded both the Introduction and Discussion to more comprehensively discuss the established context-dependent roles of IL-22 during other viral infections, while emphasizing that our study identifies a novel function for IL-22 specifically in the biology of chronic gammaherpesvirus infection. We believe these revisions more accurately reflect both the existing literature and the novelty of our findings.

Minor comment 4

Line 15: should be improved.

Response: We thank the reviewer for pointing this out. In response, we have revised the text in the Abstract (Line 15 in the original submission) to improve its clarity, readability, and overall flow. The revised wording more accurately reflects the scope of our study and is better aligned with the conclusions supported by our data.

Minor comment 5

Line 41: typo.

Response: We thank the reviewer for identifying this typographical error. The error has been corrected in the revised manuscript.

Suggestions made by reviewer 1 and response

The paper would be much stronger if the following experiments were added:

1. At early infection (e.g., 3–7 dpi), measure viral titers in lung, and measure viral genome copy number. This would determine whether IL-22 affects early replication.

Response: We thank the reviewer for this excellent suggestion and agree that distinguishing the role of IL-22 during acute lytic infection from its role in the establishment of chronic infection was an important question. In response, we performed additional experiments to directly assess acute MHV68 replication in the lungs, the primary site of lytic infection following intranasal inoculation.

Specifically, we quantified infectious viral titers by plaque assay and viral lytic/early gene ORF9 levels by quantitative PCR at 7 and 9 days post-infection (dpi), corresponding to the peak of acute lytic replication and the onset of viral clearance, respectively (new Fig. 2A–D). We found that both infectious viral titers and viral genome levels were comparable between MHV68-infected BL6 and IL-22$^{-/-}$ mice at both time points, demonstrating that IL-22 deficiency does not impair acute viral replication.

These new findings exclude the possibility that the reduced latent viral burden observed in IL-22-deficient mice is simply a consequence of diminished early viral replication. Instead, they support our conclusion that IL-22 primarily promotes the establishment of chronic gammaherpesvirus infection rather than the acute lytic phase of infection. The Results, Discussion, Figure 2, Figure 2 legend, and Materials and Methods sections have been revised accordingly.

2. Identify IL-22 target cells. The authors can consider IL-22Rα expression analysis, stromal cell analysis, and conditional IL-22R knockout.

Response: We thank the reviewer for this insightful suggestion and agree that identifying the IL-22-responsive cellular compartment would provide important mechanistic insight into how IL-22 promotes chronic gammaherpesvirus infection. We also agree that approaches such as analysis of IL-22Rα expression and the use of cell type-specific IL-22Rα-deficient mice represent the most rigorous strategy to define the relevant IL-22-responsive cell population.

Although these studies are beyond the scope of the present work, we have substantially strengthened the mechanistic framework of the manuscript through several additional experiments. Specifically, we performed new analyses of BAFF, CXCL12, and CXCL13, molecules known to regulate B-cell survival, lymphoid organization, and germinal center maintenance (new Fig. 7). These studies identified significantly reduced systemic BAFF levels

in IL-22-deficient mice, providing a potential link between IL-22 signaling and the impaired germinal center response observed during chronic infection.

In addition, we have extensively revised the Discussion to more clearly describe the likely indirect nature of IL-22 signaling. We now discuss that, because IL-22Rα expression is largely restricted to non-hematopoietic cells, IL-22 is unlikely to act directly on B cells and instead may influence germinal center responses through IL-22-responsive stromal or other non-hematopoietic cells within the lymphoid microenvironment. We further acknowledge that identifying the precise IL-22-responsive cellular target and downstream signaling pathway will require future studies using conditional IL-22Rα deletion models.

Accordingly, we have moderated our mechanistic conclusions throughout the manuscript to ensure they remain fully supported by the available data.

3. Cytokine/chemokine analysis in germinal centers including CXCL13, IL-21 and BAFF. This could explain how IL-22 supports germinal center expansion.

Response: We thank the reviewer for this valuable suggestion. To further investigate potential mechanisms underlying the impaired germinal center response observed in IL-22-deficient mice, we performed additional analyses of circulating BAFF, CXCL12, and CXCL13, key regulators of B-cell survival, lymphoid organization, and germinal center maintenance (new **Fig. 7**).

Our analyses revealed a significant reduction in systemic BAFF levels in MHV68-infected IL-22$^{-/-}$ mice. Given the well-established role of BAFF in promoting B-cell survival, activation, and differentiation, these findings provide a potential mechanistic link between IL-22 signaling and the impaired germinal center response observed during chronic infection.

Consistent with these observations, IL-22-deficient mice also exhibited reduced germinal center B cells, T follicular helper cells, plasma cells, age-associated B cells, and diminished virus-specific and polyclonal antibody responses. Together, these new findings substantially strengthen the mechanistic framework of the study and support a role for IL-22 in promoting the immune microenvironment that facilitates germinal center responses during chronic MHV68 infection. The Results, Discussion, Figure 7, and corresponding Materials and Methods sections have been revised accordingly.

4. Even though B cells lack IL-22R, IL-22 could indirectly affect them via follicular dendritic cells and stromal cells. Demonstrating this would provide mechanistic depth.

Response: We thank the reviewer for this insightful suggestion and agree that indirect signaling through IL-22-responsive cells within the lymphoid microenvironment represents the most plausible mechanism by which IL-22 influences germinal center responses. Since B cells do not express the functional IL-22 receptor, we likewise believe that the observed phenotype is unlikely to result from direct IL-22 signaling to B cells.

Although direct investigation of follicular dendritic cells or stromal cell-specific IL-22 signaling is beyond the scope of the present study, we have strengthened the mechanistic framework of the manuscript in several ways. First, we performed additional analyses of BAFF, CXCL12, and CXCL13, molecules involved in B-cell survival, lymphoid organization, and germinal center maintenance. These studies identified significantly reduced systemic BAFF levels in IL-22-deficient mice, providing a potential link between IL-22 signaling and the impaired germinal center response.

Second, we have substantially expanded the Discussion to emphasize that IL-22 most likely acts indirectly through IL-22-responsive non-hematopoietic cells within the lymphoid microenvironment. We now discuss stromal cells as plausible intermediaries that could influence germinal center formation and B-cell differentiation, while explicitly acknowledging that defining the precise IL-22-responsive cellular target will require future studies employing cell type-specific IL-22Rα deletion models.

We believe these revisions provide a more balanced mechanistic interpretation while appropriately recognizing the limitations of the current study.

Reviewer 2

Comment summary

In this manuscript by Majeed et al, the authors show that IL-22 is required for establishment of murine gammaherpsvirus-68 (MHV68) latency. They also show that virus-specific CD8 T cells, germinal center B cells and T follicular helper cells are reduced in IL-22 KO mice. When they measured the antibody response, they found that KO mice produced equivalent amounts of anti-MHV68 antibody, but had reduced production of autoantibodies (anti-ANA, anti-dsDNA). Overall, the data supporting reduced MHV68 latency and reduced T cell and germinal center B cell responses is convincing. However, there is no mechanistic link between IL-22 and support of viral latency. In fact, IL-22 receptor is not expressed on B cells, so how IL-22 is supporting

viral latency in B cells is a major knowledge gap. The lack of mechanistic insight and the points listed below, dampen enthusiasm for the paper.

Response: We sincerely thank the reviewer for the careful evaluation of our manuscript and for recognizing the strength of our data demonstrating reduced MHV68 latency together with diminished virus-specific $CD8^+$ T-cell, germinal center B-cell, and T follicular helper cell responses in IL-22-deficient mice. We appreciate the reviewer's thoughtful comments regarding the mechanistic basis of IL-22 function during chronic gammaherpesvirus infection.

In response to the reviewer's suggestions, we have substantially strengthened the manuscript through extensive new experiments and major revisions. These include analyses of acute viral replication to distinguish effects on lytic versus latent infection, expanded characterization of IL-22-producing immune cell populations, mechanistic analyses of BAFF, CXCL12, and CXCL13, additional characterization of activated B-cell subsets, expanded analyses of virus-specific and autoreactive antibody responses, and substantial revisions to the Discussion. We have also moderated our mechanistic conclusions throughout the manuscript and now more clearly discuss the likely indirect role of IL-22 signaling through the lymphoid microenvironment, while acknowledging that identification of the IL-22-responsive cellular target will require future investigation.

We believe these revisions substantially strengthen the mechanistic framework of the study while remaining appropriately aligned with the conclusions supported by our data. Our detailed responses to each comment are provided below.

Comment 1

Given the reduction in latent viral load and reduced virus-specific CD8 T cells, analysis of acute replication of MHV68 in the lung, spleen, and peritoneal cavity is also needed. The lack of robust virus-specific CD8 T cell expansion in the spleen is more likely due to less replication at acute timepoints rather than less latency at day 16. It is also possible that replication in the spleen but not the peritoneal cavity is reduced, which would explain why virus-specific CD8 T cells in WT and KO are equivalent in the peritoneal cavity.

Response: We thank the reviewer for this thoughtful comment and agree that distinguishing the effects of IL-22 on acute viral replication from its role in the establishment of chronic infection was essential for interpreting the reduced virus-specific $CD8^+$ T-cell response observed in IL-22-deficient mice.

To address this concern, we performed additional experiments examining acute MHV68 replication in the lungs, the primary site of lytic infection following intranasal inoculation. Specifically, we quantified infectious viral titers by plaque assay and viral genome levels by quantitative PCR at 7 and 9 days post-infection (dpi), corresponding to the peak of acute lytic replication and the onset of viral clearance, respectively (new Fig. 2A–D). These analyses revealed no significant differences between BL6 and IL-22$^{-/-}$ mice, demonstrating that IL-22 deficiency does not impair acute viral replication.

In addition, we expanded our analysis of virus-specific CD8^{+} T-cell responses and now show that, in contrast to our original submission, virus-specific CD8^{+} T cells are significantly reduced in both the spleen and peritoneal cavity of IL-22-deficient mice during chronic infection (new Fig. 4). Despite this reduction, global CD8^{+} T-cell activation, IFNγ production by splenic virus-specific CD8^{+} T cells, and systemic inflammatory cytokine levels remained comparable between genotypes (new Fig. 5).

Together, these findings indicate that the diminished virus-specific CD8^{+} T-cell response cannot be explained by impaired acute viral replication. Instead, our data support the interpretation that IL-22 primarily promotes the establishment of chronic gammaherpesvirus infection, with the reduced virus-specific CD8^{+} T-cell response likely reflecting diminished antigenic stimulation associated with the smaller latent viral reservoir.

Comment 2

Along with measuring acute replication of MHV68, levels of IL-22 should also be determined at those same timepoints.

Response: We thank the reviewer for this valuable suggestion and agree that characterizing IL-22 expression during the transition from acute to chronic infection provides important biological context for interpreting its role during MHV68 infection.

In response, we expanded our temporal analysis of IL-22 expression and quantified IL-22 protein levels in the lungs, spleen, and serum at 9 days post-infection (dpi), corresponding to the transition from acute lytic infection toward the establishment of latency, as well as at 16 dpi, the peak of latent infection (new Fig. 1A–F). These analyses demonstrated that IL-22 production is induced early during infection, with elevated levels detected in the lungs at 9 dpi, followed by a significant increase in the spleen and serum at 16 dpi as chronic infection becomes established.

Together with our new acute viral replication data (new Fig. 2), these findings indicate that IL-22 is induced during the early phase of infection despite having no detectable effect on acute viral replication, supporting the conclusion that IL-22 primarily contributes to the establishment of chronic gammaherpesvirus infection rather than the lytic phase of infection. The Results, Discussion, Figure 1, and corresponding figure legend have been revised accordingly.

Comment 3

The statement made in line 158-159 should be modified. The authors claim that IL-22 is required for latency establishment and the capacity to reactivate. However, the defect in reactivation is most likely because there is less latent viral load and not because there is actually a defect in the capacity to reactivate. The fact that the difference between WT and KO does not increase between d16 and d42, suggests that the virus can reactivate in IL-22 KO mice comparable to WT.

Response: We thank the reviewer for this insightful comment and agree with this interpretation. We acknowledge that the reduced frequency of ex vivo viral reactivation observed in IL-22$^{-/-}$ mice is most appropriately interpreted as a consequence of the reduced latent viral reservoir rather than an intrinsic defect in the ability of latently infected cells to reactivate.

Accordingly, we have revised the Results and Discussion throughout the manuscript to clarify this distinction. Specifically, we no longer state that IL-22 regulates the "capacity to reactivate." Instead, we now interpret the reduced ex vivo reactivation frequency in IL-22-deficient mice as reflecting the diminished number of latently infected cells available to undergo reactivation.

To further strengthen this interpretation, we extended our analyses to 42 days post-infection, demonstrating that although IL-22$^{-/-}$ mice continue to harbor a significantly smaller latent viral reservoir than BL6 mice, the magnitude of this difference remains comparable to that observed at 16 days post-infection. These findings are consistent with the reviewer's interpretation and indicate that IL-22 primarily promotes the establishment of the latent reservoir rather than directly regulating the intrinsic reactivation capacity of latently infected cells.

The Results and Discussion have been revised accordingly to more accurately reflect these conclusions.

Comment 4

If IL-22 is required to support a germinal center response, then it is unclear why anti-MHV68 antibody levels are unaffected by loss of IL-22. Rather than a quantitative difference in total anti-MHV68 antibody, is there a qualitative difference in the anti-MHV68 antibody? Throughout, the authors describe a 'profound reduction in the virus-driven germinal center response', which was shown as reduced germinal center B cells and fewer T follicular helper cells, but if the germinal center response is profoundly impacted by loss of IL-22, then why isn't anti-MHV68 antibody response negatively impacted?

Response: We thank the reviewer for this insightful comment. We agreed that the preservation of virus-specific antibody responses despite a diminished germinal center response represented an important issue that warranted further investigation.

To address this concern, we expanded our analysis of the humoral immune response by performing additional experiments and increasing the number of animals analyzed. These studies demonstrated that, contrary to our original observations, IL-22-deficient mice exhibit a significant reduction in both MHV68-specific IgG and MHV68-specific IgM antibody responses during peak chronic infection (16 dpi) (new Fig. 9C–D). These findings are consistent with the observed reductions in germinal center B cells, T follicular helper cells, plasma cells, and systemic BAFF levels, thereby establishing a coherent relationship between the impaired germinal center response and diminished antiviral humoral immunity.

To further determine whether these defects persisted during long-term infection, we additionally examined humoral responses at **42 dpi**. At this later stage, total immunoglobulin levels, MHV68-specific antibody responses, and autoreactive antibody production were comparable between BL6 and IL-22$^{-/-}$ mice, paralleling the normalization of germinal center responses observed at the same time point (new **Fig. 6** and **Fig. 9**).

Accordingly, the Results and Discussion have been extensively revised to incorporate these new findings. We believe these additional experiments resolve the apparent discrepancy identified by the reviewer and substantially strengthen the relationship between IL-22, germinal center responses, and virus-specific humoral immunity.

Comment 5

The title says that IL-22 supports pathogenesis, but there is not any data in the paper related to pathogenesis, per se.

Response: We thank the reviewer for this helpful suggestion and agree that the term "pathogenesis" was too broad, as the present study primarily investigates the role of IL-22 in the establishment of chronic gammaherpesvirus infection rather than disease pathogenesis per se.

Accordingly, we have revised the title to more accurately reflect the scope and conclusions of the study. The revised title is:

"IL-22 promotes the establishment of chronic gammaherpesvirus infection by supporting the germinal center response." We believe this revised title more precisely captures the principal findings of the manuscript and avoids overstating the implications of our data.

Reviewer 3

The work in this manuscript addresses the role of host cytokine IL-22 in gammaherpesvirus infection. IL-22 is well known to act on epithelial cells and stromal cells to promote antimicrobial defenses at mucosal barriers. It is also been shown to be important in tissue regeneration in some scenarios and in promoting tertiary lymphoid organ development. However, its specific function in gammaherpesvirus infections and B cells has not been specifically tested. That said, it is notable that IL-22 is a member of the same family as IL-10, an immunosuppressive cytokine that is well known to promote gammaherpesvirus infection in B cells through virus induction of host IL-10 or through virus expression of a viral IL-10 homolog. Here, the authors examine the function of IL-22, for both the virus and the host immune response, during murine gammaherpesvirus (MHV68) infection. On the virus side, they find that lack of IL-22 results in decreased latency establishment, suggesting that the cytokine is proviral. On the host side, they determine that the virus strongly induces an IL-22 response in CD4 and CD8 T cells, and that IL-22-deficient mice exhibit impaired expansion of CD8 T cells, significantly reduced germinal center B cell expansion, and reduced polyclonal and autoantibody responses. Although the manuscript is by necessity slanted toward a virology audience, perhaps the most important aspect of this manuscript is the contribution of IL-22 to germinal center expansion and autoantibody responses. This is a unique finding that could impact the response to multiple pathogens as well our understanding of antibody-mediated autoimmunity. While the effect of IL-22 on promoting virus infection is modest, the results are consistent with the authors' demonstration of the ability of IL-22 to promote germinal center reactions.

Response: We sincerely thank the reviewer for the thoughtful and highly encouraging evaluation of our manuscript. We greatly appreciate the reviewer's recognition of the novelty of our findings and the insightful observation that the impact of IL-22 on germinal center biology

and humoral immunity may extend beyond gammaherpesvirus infection to broader aspects of host immunity and antibody-mediated autoimmunity. We are also grateful for the reviewer's positive assessment that the observed effects on viral latency are biologically consistent with the diminished germinal center response.

In response to the reviewer's comments, we have further strengthened the manuscript through several additional experiments, including analyses of acute viral replication, expanded characterization of IL-22-producing immune cell populations, additional analyses of virus-specific $CD8^+$ T-cell responses, mechanistic studies of BAFF, CXCL12, and CXCL13, expanded characterization of activated B-cell subsets, and additional analyses of virus-specific and autoreactive antibody responses. We have also substantially revised the Discussion to better define the mechanistic framework of the study while moderating our conclusions where appropriate.

We appreciate the reviewer's constructive suggestions, which have significantly improved the quality and clarity of the manuscript. Our detailed responses are provided below.

Comment 1

For completion sake, it would be useful to show the impact of IL-22 on the acute stage of virus infection (eg, virus titers in lung). This is particular the case because early decreases in titers could delay latency establishment and T cell responses.

Response: We thank the reviewer for this excellent suggestion and agree that evaluation of acute viral replication is important to distinguish whether the reduced latent viral burden and diminished virus-specific T-cell responses observed in IL-22-deficient mice are secondary to impaired early infection.

To address this concern, we performed additional experiments examining acute MHV68 replication in the lungs, the primary site of lytic infection following intranasal inoculation. Specifically, we quantified infectious viral titers by plaque assay together with viral genome levels by quantitative PCR at 7 and 9 days post-infection (dpi), corresponding to the peak of acute lytic replication and the onset of viral clearance, respectively (new Fig. 2A–D). These analyses demonstrated that acute viral replication was comparable between BL6 and IL-$22^{-/-}$ mice at both time points.

These new findings indicate that IL-22 deficiency does not impair acute MHV68 replication and therefore exclude delayed acute infection as an explanation for the reduced latent viral burden

and diminished virus-specific CD8$^+$ T-cell responses observed during chronic infection. Instead, the data support a role for IL-22 in promoting the establishment of chronic gammaherpesvirus infection. The Results, Discussion, Figure 2, Figure 2 legend, and Materials and Methods sections have been revised accordingly.

Comment 2

What are the frequencies of latently infected cells in the assays in Fig 2? The latency results shown clear trend toward a phenotype, but the assays themselves (and the limiting dilution PCR assay) seem as though they need some technical improvement. Shouldn't percent reactions positive decrease significantly toward zero over the course of the dilution series? This does not seem to be the case with these results (especially panel C and E), which makes interpretation of frequencies of infection impossible. In addition, any interpretation that IL-22 effects both latency and reactivation from latency should be supported with calculations of frequencies of both. ie, if there is reduced latently infected cells, shouldn't there also be reduced reactivation from latency? If that is the case then IL-22 may support latency establishment, but have no real effect on reactivation itself.

Response: We thank the reviewer for this important comment. We agree that the frequencies of cells harboring MHV68 genome and cells capable of ex vivo reactivation should be clearly reported to support interpretation of the limiting dilution assays.

To address this concern, we have added Table 1 to the revised manuscript, which reports the calculated frequencies of MHV68 genome-positive cells and cells exhibiting ex vivo reactivation in the spleen and peritoneal cavity at the indicated time points. We believe this addition improves the clarity and interpretability of the latency and reactivation data.

We also agree with the reviewer that reduced ex vivo reactivation is most appropriately interpreted in the context of the reduced frequency of latently infected cells. Accordingly, we have revised the Results and Discussion to avoid stating that IL-22 directly regulates the intrinsic capacity of latent virus to reactivate. Instead, we now interpret the reduced ex vivo reactivation frequency in IL-22-deficient mice as likely reflecting the smaller latent viral reservoir available to undergo reactivation.

Thus, the revised manuscript now supports the conclusion that IL-22 promotes the establishment of the latent MHV68 reservoir, while avoiding overinterpretation of the ex vivo reactivation data as evidence for a direct defect in reactivation capacity.

Comment 3

It would be highly worthwhile and a strong addition to the manuscript (but perhaps outside the scope of the current manuscript) to examine more mechanistically why GC B cells are expanded in response to IL-22. For example, if the effects are not directly on GC B cells, then is the stromal microenviroment altered in some way?

Response: We thank the reviewer for this insightful suggestion and agree that defining the mechanism by which IL-22 promotes germinal center expansion represents an important next step. We also appreciate the reviewer's recognition that these studies may be beyond the scope of the present manuscript.

Although we did not directly investigate the stromal microenvironment or identify the IL-22-responsive cellular target, we have substantially strengthened the mechanistic framework of the manuscript through several additional experiments. Specifically, we quantified serum levels of BAFF, CXCL12, and CXCL13, key regulators of B-cell survival, lymphoid organization, and germinal center maintenance (new **Fig. 7**). These studies revealed significantly reduced systemic BAFF levels in IL-22-deficient mice, providing a potential link between IL-22 signaling and the impaired germinal center response.

In addition, we have substantially expanded the Discussion to more clearly describe the likely indirect nature of IL-22 signaling. We now discuss that, because IL-22Rα expression is largely restricted to non-hematopoietic cells, IL-22 is unlikely to act directly on germinal center B cells but instead may influence germinal center responses through IL-22-responsive stromal or other non-hematopoietic cells within the lymphoid microenvironment. We further acknowledge that defining the precise IL-22-responsive cellular target will require future studies using cell type-specific IL-22Rα deletion models.

We believe these additional experiments and revisions provide a stronger mechanistic framework while appropriately recognizing the limitations of the current study.

Minor comment 1

Considering the modest results of the latency assays, the statement at the end of the abstract that IL-22 may be “a potential therapeutic target for disrupting the establishment of lifelong viral latency and its associated diseases” seems overstated.

Response: We thank the reviewer for this thoughtful comment and agree that our original statement overstated the translational implications of the findings. Accordingly, we have revised the final sentence of the Abstract to moderate our conclusions. Similar revisions have also been made throughout the Discussion to ensure that the translational implications remain appropriately aligned with the scope of the current study.

We believe these revisions more accurately reflect the significance of our findings while avoiding overinterpretation of their potential therapeutic implications.

Minor comment 2

Line 156: post-challenge? Or post-plating?

Response: We thank the reviewer for identifying this ambiguity. The reviewer is correct that the time indicated refers to **days post-plating** of splenocytes/peritoneal cells onto mouse embryonic fibroblast (MEF) monolayers for the ex vivo reactivation assay, rather than days post-challenge (infection). We have corrected the text in the revised manuscript to accurately reflect this experimental procedure and improve clarity.

Minor comment 3.

The authors should avoid using the word "significant" in the text with regard to data that have no statistical support for significance. (eg, with regard to Fig 2 data).

Response: We thank the reviewer for this important observation and agree that non-significant findings should not be described using language that implies statistical significance.

In response, we carefully reviewed the entire manuscript and revised the text throughout the Results and Discussion to ensure that all observations are described in a manner consistent with their statistical support. Specifically, statements referring to non-significant differences have been revised to indicate that the data "trended higher," "trended lower," or "did not reach statistical significance," where appropriate. This includes the latency and ex vivo reactivation data presented in Figure 3 (formerly Fig. 2), as well as other instances throughout the manuscript.

We believe these revisions provide a more accurate and balanced presentation of the data and ensure that our interpretations are fully supported by the statistical analyses.

Minor comment 4.

Line 166: if these are experiments that were performed but data are not shown, this should be stated clearly.

Minor comment 5.

5. Line 203: how do the authors reconcile decreased absolute numbers of T cells with no change in splenomegaly?

Response: We thank the reviewer for this insightful question. We agree that this point required further clarification. MHV68-induced splenomegaly reflects the overall lymphoproliferative expansion of multiple immune cell populations during infection and is therefore distinct from changes occurring within specific activated lymphocyte subsets.

To address this point, we expanded our analyses of splenic pathology in the revised manuscript by measuring gross splenic morphology, spleen weights, total splenocyte numbers, and total $B220^+$ B-cell numbers. These analyses demonstrated that IL-22 deficiency does not significantly alter overall splenic enlargement or total lymphocyte accumulation during chronic MHV68 infection (new **Fig. 6A–D**). In contrast, the reductions observed in IL-22-deficient mice were restricted to specific activated populations, including germinal center B cells, T follicular helper cells, plasma cells, and age-associated B cells.

Accordingly, we have revised the Results and Discussion to clarify that IL-22 deficiency does not impair the overall lymphoproliferative response responsible for splenomegaly, but rather selectively affects virus-driven activated B-cell and T-cell subsets that support chronic infection. We believe these revisions resolve the apparent discrepancy identified by the reviewer.

Minor Comment 6

The authors have a section in the Discussion about how IL-22 sustains chronic infection, but don’t actually say much other than it must act on non-B cells. Because this effect on B cells is such a centrally important issue for this manuscript, further discussion is warranted here.

Response: We thank the reviewer for this thoughtful suggestion and agree that a more comprehensive discussion of the potential mechanisms linking IL-22 signaling to germinal center biology would strengthen the manuscript.

In response, we have substantially revised and expanded the Discussion section entitled **"How IL-22 Sustains Chronic Infection."** To further strengthen this mechanistic framework, we

incorporated new experimental findings demonstrating significantly reduced systemic BAFF levels in IL-22-deficient mice together with analyses of CXCL12 and CXCL13.

We believe these revisions provide a more comprehensive and balanced discussion of the potential mechanisms underlying the observed phenotype.
