# Supplementary material for: IL-22 promotes the establishment of chronic gammaherpesvirus infection by supporting the germinal center response
Source: PLoS Pathog. 2026 Sep 8;22(9):e1014016. doi: 10.1371/journal.ppat.1014016 (PMC13585334; doi:10.1371/journal.ppat.1014016)
Supplement: Peer review history

# Peer review: Decision Letter 0

**DOI:** 10.1371/journal.ppat.1014016.r001

**Authors:** Donna M Neumann, Italo Tempera

25 Mar 2026

IL-22 Promotes Gammaherpesvirus Latency and Pathogenesis by Supporting Germinal Center Expansion and Polyclonal Autoimmunity

PLOS Pathogens

Dear Dr. Jondle,

Thank you for submitting your manuscript to PLOS Pathogens. After careful consideration, we feel that it has merit but does not fully meet PLOS Pathogens's publication criteria as it currently stands. Therefore, we invite you to submit a revised version of the manuscript that addresses the points raised during the review process.

Please submit your revised manuscript by May 24 2026 11:59PM. If you will need more time than this to complete your revisions, please reply to this message or contact the journal office at plospathogens@plos.org. When you're ready to submit your revision, log on to https://www.editorialmanager.com/ppathogens/ and select the 'Submissions Needing Revision' folder to locate your manuscript file.

Please include the following items when submitting your revised manuscript:

* A letter that responds to each point raised by the editor and reviewer(s). You should upload this letter as a separate file labeled 'Response to Reviewers'. This file does not need to include responses to any formatting updates and technical items listed in the 'Journal Requirements' section below.

* A marked-up copy of your manuscript that highlights changes made to the original version. You should upload this as a separate file labeled 'Revised Manuscript with Track Changes'.

* An unmarked version of your revised paper without tracked changes. You should upload this as a separate file labeled 'Manuscript'.

If you would like to make changes to your financial disclosure, competing interests statement, or data availability statement, please make these updates within the submission form at the time of resubmission. Guidelines for resubmitting your figure files are available below the reviewer comments at the end of this letter.

We look forward to receiving your revised manuscript.

Kind regards,

Italo Tempera, PHD

Academic Editor

PLOS Pathogens

Donna Neumann

Section Editor

PLOS Pathogens

Editor-in-Chief

PLOS Pathogens

orcid.org/0000-0003-2946-9497

Editor-in-Chief

PLOS Pathogens

orcid.org/0000-0002-7699-2064

**Journal Requirements:**

1) Please ensure that the CRediT author contributions listed for every co-author are completed accurately and in full.

At this stage, the following Authors/Authors require contributions: Sheikh Tahir Majeed, Samantha M. Bradford, and Christopher Jondle. Please ensure that the full contributions of each author are acknowledged in the "Add/Edit/Remove Authors" section of our submission form.

The list of CRediT author contributions may be found here: https://journals.plos.org/plospathogens/s/authorship#loc-author-contributions

2) We do not publish any copyright or trademark symbols that usually accompany proprietary names, eg ©,  ®, or TM  (e.g. next to drug or reagent names). Therefore please remove all instances of trademark/copyright symbols throughout the text, including:

- TM on page: 20.

3) Please add a full list of legends for your Supporting Information files in the manuscript after the references list.

4) When completing the data availability statement of the submission form, you indicated that you will make your data available on acceptance. We strongly recommend all authors decide on a data sharing plan before acceptance, as the process can be lengthy and hold up publication timelines. Please note that, though access restrictions are acceptable now, your entire data will need to be made freely accessible if your manuscript is accepted for publication. This policy applies to all data except where public deposition would breach compliance with the protocol approved by your research ethics board.

Note: If the reviewer comments include a recommendation to cite specific previously published works, please review and evaluate these publications to determine whether they are relevant and should be cited. There is no requirement to cite these works unless the editor has indicated otherwise.

**Reviewers' Comments:**

Reviewer's Responses to Questions

**Part I - Summary**

Please use this section to discuss strengths/weaknesses of study, novelty/significance, general execution and scholarship.

Reviewer #1: This manuscript investigates the role of IL-22 in murine gammaherpesvirus 68 (MHV68) infection, demonstrating that IL-22 promotes viral latency, germinal center expansion, and polyclonal antibody production. Using IL-22 knockout mice, the authors report reduced latent viral load, impaired reactivation, diminished germinal center responses, and reduced autoantibody production. The study proposes that IL-22 acts as a host factor exploited by gammaherpesviruses to support chronic infection.

The work addresses an interesting question regarding host cytokines that support viral latency, which is relevant to EBV/KSHV biology. In particular, the role of IL-22 in gammaherpesvirus latency has not been extensively explored and represents a potentially novel host pathway influencing viral persistence. The authors examine IL-22 induction after infection, latency frequency, viral reactivation, germinal center responses, CD8 T cell responses and antibody production. These works provide a broad view of how IL-22 deficiency alters infection. The reduced germinal center response correlates well with reduced latency and reduced polyclonal antibody response, which support the overall model. However, while the study is solid at the descriptive level, the mechanistic depth is limited, and several conclusions are not sufficiently supported by the data.

Overall, the manuscript is potentially suitable for PLOS Pathogens, but major revisions are required to strengthen mechanistic insight and address several conceptual issues.

Major Concerns:

The central conclusion is that IL-22 promotes viral latency by supporting germinal center expansion. However, the study does not determine which cells respond to IL-22? how IL-22 signaling promotes germinal center formation? what downstream pathways are involved?

The manuscript itself notes that B cells lack IL-22Rα, implying the effect must be indirect. But no experiments were done to address stromal cells, follicular dendritic cells, epithelial cells, and cytokine/chemokine changes. Thus, the mechanistic explanation remains speculative.

The authors interpret IL-22 as a proviral factor promoting latency, but an alternative explanation is not excluded. Specifically, IL-22 may enhance early viral replication, which then secondarily increases the latent reservoir. The manuscript does not measure acute viral titers, early infection levels and lytic replication. These data are necessary before concluding IL-22 specifically promotes latency.

The observed germinal center defect may simply reflect reduced antigen load. The authors’ interpretation is that IL-22 affects germinal center expansion, which then affect latency. However, the reverse is equally plausible: Reduced viral burden could lead to reduced antigen, which could reduced germinal centers. Importantly, the CD8 T cell data already suggest reduced antigen load in IL-22 knockout mice. Thus, the causal direction remains unclear.

The manuscript states that virus-specific CD4 and CD8 T cells produce IL-22. However, IL-22 can also originate from ILC3 cells, γδ T cells and Th17 cells. A clearer characterization of the cellular sources would strengthen the study.

The manuscript frequently references EBV and KSHV, but all data are from MHV68 infection in mice. There is no evidence that IL-22 affects EBV latency and IL-22 affects KSHV infection. Some supporting evidence from human datasets or literature would strengthen the translational significance.

Minor Concerns:

The manuscript contains several redundant statements. The Discussion could be significantly condensed.

Some figures report trends without clear statistical support (e.g., IL-22 levels in spleen at early time points). Statistical methods should be more clearly described.

The manuscript states that data hosting is still being determined. PLOS requires data availability upon publication.

The authors state “first evidence that IL-22 plays a proviral role in gammaherpesvirus infection”. This should be toned down slightly. While the specific context may be new, IL-22 has already been implicated in viral pathogenesis in other infections.

Line 15: should be improved.

Line 41: typo.

Reviewer #2: In this manuscript by Majeed et al, the authors show that IL-22 is required for establishment of murine gammaherpsvirus-68 (MHV68) latency. They also show that virus-specific CD8 T cells, germinal center B cells and T follicular helper cells are reduced in IL-22 KO mice. When they measured the antibody response, they found that KO mice produced equivalent amounts of anti-MHV68 antibody, but had reduced production of autoantibodies (anti-ANA, anti-dsDNA). Overall, the data supporting reduced MHV68 latency and reduced T cell and germinal center B cell responses is convincing. However, there is no mechanistic link between IL-22 and support of viral latency. In fact, IL-22 receptor is not expressed on B cells, so how IL-22 is supporting viral latency in B cells is a major knowledge gap. The lack of mechanistic insight and the points listed below, dampen enthusiasm for the paper.

Reviewer #3: The work in this manuscript addresses the role of host cytokine IL-22 in gammaherpesvirus infection. IL-22 is well known to act on epithelial cells and stromal cells to promote antimicrobial defenses at mucosal barriers. It is also been shown to be important in tissue regeneration in some scenarios and in promoting tertiary lymphoid organ development. However, its specific function in gammaherpesvirus infections and B cells has not been specifically tested. That said, it is notable that IL-22 is a member of the same family as IL-10, an immunosuppressive cytokine that is well known to promote gammaherpesvirus infection in B cells through virus induction of host IL-10 or through virus expression of a viral IL-10 homolog. Here, the authors examine the function of IL-22, for both the virus and the host immune response, during murine gammaherpesvirus (MHV68) infection. On the virus side, they find that lack of IL-22 results in decreased latency establishment, suggesting that the cytokine is proviral. On the host side, they determine that the virus strongly induces an IL-22 response in CD4 and CD8 T cells, and that IL-22-deficient mice exhibit impaired expansion of CD8 T cells, significantly reduced germinal center B cell expansion, and reduced polyclonal and autoantibody responses. Although the manuscript is by necessity slanted toward a virology audience, perhaps the most important aspect of this manuscript is the contribution of IL-22 to germinal center expansion and autoantibody responses. This is a unique finding that could impact the response to multiple pathogens as well our understanding of antibody-mediated autoimmunity. While the effect of IL-22 on promoting virus infection is modest, the results are consistent with the authors’ demonstration of the ability of IL-22 to promote germinal center reactions.

**********

**Part II – Major Issues: Key Experiments Required for Acceptance**

Please use this section to detail the key new experiments or modifications of existing experiments that should be absolutely required to validate study conclusions.

Generally, there should be no more than 3 such required experiments or major modifications for a "Major Revision" recommendation. If more than 3 experiments are necessary to validate the study conclusions, then you are encouraged to recommend "Reject".

Reviewer #1: Experiments that would greatly strengthen the manuscript:

The paper would be much stronger if the following experiments were added:

At early infection (e.g., 3–7 dpi), measure viral titers in lung, and measure viral genome copy number. This would determine whether IL-22 affects early replication.

Identify IL-22 target cells. The authors can consider IL-22Rα expression analysis, stromal cell analysis, and conditional IL-22R knockout.

Cytokine/chemokine analysis in germinal centers including CXCL13, IL-21 and BAFF. This could explain how IL-22 supports germinal center expansion.

Even though B cells lack IL-22R, IL-22 could indirectly affect them via follicular dendritic cells and stromal cells. Demonstrating this would provide mechanistic depth.

Reviewer #2: 1. Given the reduction in latent viral load and reduced virus-specific CD8 T cells, analysis of acute replication of MHV68 in the lung, spleen, and peritoneal cavity is also needed. The lack of robust virus-specific CD8 T cell expansion in the spleen is more likely due to less replication at acute timepoints rather than less latency at day 16. It is also possible that replication in the spleen but not the peritoneal cavity is reduced, which would explain why virus-specific CD8 T cells in WT and KO are equivalent in the peritoneal cavity.

2. Along with measuring acute replication of MHV68, levels of IL-22 should also be determined at those same timepoints.

3. The statement made in line 158-159 should be modified. The authors claim that IL-22 is required for latency establishment and the capacity to reactivate. However, the defect in reactivation is most likely because there is less latent viral load and not because there is actually a defect in the capacity to reactivate. The fact that the difference between WT and KO does not increase between d16 and d42, suggests that the virus can reactivate in IL-22 KO mice comparable to WT.

4. If IL-22 is required to support a germinal center response, then it is unclear why anti-MHV68 antibody levels are unaffected by loss of IL-22. Rather than a quantitative difference in total anti-MHV68 antibody, is there a qualitative difference in the anti-MHV68 antibody? Throughout, the authors describe a ‘profound reduction in the virus-driven germinal center response’, which was shown as reduced germinal center B cells and fewer T follicular helper cells, but if the germinal center response is profoundly impacted by loss of IL-22, then why isn’t anti-MHV68 antibody response negatively impacted?

5. The title says that IL-22 supports pathogenesis, but there is not any data in the paper related to pathogenesis, per se.

Reviewer #3: 1. For completion sake, it would be useful to show the impact of IL-22 on the acute stage of virus infection (eg, virus titers in lung). This is particular the case because early decreases in titers could delay latency establishment and T cell responses.

2. What are the frequencies of latently infected cells in the assays in Fig 2? The latency results shown clear trend toward a phenotype, but the assays themselves (and the limiting dilution PCR assay) seem as though they need some technical improvement. Shouldn’t percent reactions positive decrease significantly toward zero over the course of the dilution series? This does not seems to be the case with these results (especially panel C and E), which makes interpretation of frequencies of infection impossible. In addition, any interpretation that IL-22 effects both latency and reactivation from latency should be supported with calculations of frequencies of both. ie, if there is reduced latently infected cells, shouldn’t there also be reduced reactivation from latency? If that is the case then IL-22 may support latency establishment, but have no real effect on reactivation itself.

3. It would be highly worthwhile and a strong addition to the manuscript (but perhaps outside the scope of the current manuscript) to examine more mechanistically why GC B cells are expanded in response to IL-22. For example, if the effects are not directly on GC B cells, then is the stromal microenviroment altered in some way?

**********

**Part III – Minor Issues: Editorial and Data Presentation Modifications**

Please use this section for editorial suggestions as well as relatively minor modifications of existing data that would enhance clarity.

Reviewer #1: (No Response)

Reviewer #2: (No Response)

Reviewer #3: 1. Considering the modest results of the latency assays, the statement at the end of the abstract that IL-22 may be “a potential therapeutic target for disrupting the establishment of lifelong viral latency and its associated diseases” seems overstated.

2. Line 156: post-challenge? Or post-plating?

3. The authors should avoid using the word “significant” in the text with regard to data that have no statistical support for significance. (eg, with regard to Fig 2 data).

4. Line 166: if these are experiments that were performed but data are not shown, this should be stated clearly.

5. Line 203: how do the authors reconcile decreased absolute numbers of T cells with no change in splenomegaly?

6. The authors have a section in the Discussion about how IL-22 sustains chronic infection, but don’t actually say much other than it must act on non-B cells. Because this effect on B cells is such a centrally important issue for this manuscript, further discussion is warranted here.

**********

PLOS authors have the option to publish the peer review history of their article (what does this mean?). If published, this will include your full peer review and any attached files.

If you choose “no”, your identity will remain anonymous but your review may still be made public.

**Do you want your identity to be public for this peer review?**  For information about this choice, including consent withdrawal, please see our Privacy Policy.

Reviewer #1: No

Reviewer #2: No

Reviewer #3: No

[NOTE: If reviewer comments were submitted as an attachment file, they will be attached to this email and accessible via the submission site. Please log into your account, locate the manuscript record, and check for the action link "View Attachments". If this link does not appear, there are no attachment files.]

**Figure resubmission:**

**Reproducibility:**

?>

# Author comment: Author response to Decision Letter 1

**DOI:** 10.1371/journal.ppat.1014016.r002

16 Jul 2026

Attachment Submitted filename: Response to Reviewers.pdf

# Peer review: Decision Letter 1

**DOI:** 10.1371/journal.ppat.1014016.r003

**Authors:** Donna M Neumann, Italo Tempera

18 Aug 2026

Dear Assistant Professor Jondle,

We are pleased to inform that your manuscript, "IL-22 promotes the establishment of chronic gammaherpesvirus infection by supporting the germinal center response", has been editorially accepted for publication at PLOS Pathogens.

Before your manuscript can be formally accepted and sent to production, you will need to complete our formatting changes, which you will receive by email within a week. Please note that your manuscript will not be scheduled for publication until you have made the required changes.

IMPORTANT NOTES

(1) Copyediting and Proofreading: The corresponding author will receive a typeset proof for review, to ensure errors have not been introduced during production. Please review the PDF proof of your manuscript carefully, as this is the last chance to correct any errors. Please note that major changes, or those which affect the scientific understanding of the work, will likely cause delays to the publication date of your manuscript.

(2) Appropriate Figure Files: Please remove all name and figure # text from your figure files. Please also take this time to check that your figures are of high resolution, which will improve the readbility of your figures and help expedite your manuscript's publication. Please note that figures must have been originally created at 300dpi or higher. Do not manually increase the resolution of your files. For instructions on how to properly obtain high quality images, please review our Figure Guidelines, with examples at: http://journals.plos.org/plospathogens/s/figures.

We encourage you to use PACE (http://pace.apexcovantage.com/), a figure-checking tool which helps authors ensure all figures meet PLOS requirements so that figures can publish in the highest quality. PACE is a digital diagnostic and conversion tool for figure files. It will provide information about any failed check(s) and, if able, will automatically convert the figure into an acceptable file that passes quality checks.

To use PACE, you must first register as a user. Then, login and navigate to the UPLOAD tab, where you will find detailed instructions on how to use the tool.

(3) Striking Image: Please upload a striking still image to accompany your article if one is available (you can include a new image or an existing one from within your manuscript). Should your paper be accepted, this image will be considered for our monthly issue image and may also appear on our website to feature your article. Please upload this as a separate file, selecting "striking image" as the file type upon upload. Please also include a separate "Other" file with a caption, including credits and any potential copyright information. Please do not include the caption in the main article file. If your image is from someone other than yourself, please ensure that the artist has read and agreed to the terms and conditions of the Creative Commons Attribution License at http://journals.plos.org/plospathogens/s/content-license. Please note that PLOS cannot publish copyrighted images.

(4) Press Release or Related Media: If you or your institution will be preparing press materials for this manuscript, please inform our press team in advance at plospathogens@plos.org as soon as possible. We ask that you contact us within one week to plan ahead of our fast Production schedule. If you need to know your paper's publication date for related media purposes, you must coordinate with our press team, and your manuscript will remain under a strict press embargo until the publication date and time.

(5) Update your Profile Information: Now that your manuscript has been provisionally accepted, please log into Editorial Manager and update your profile, if needed. Go to http://www.editorialmanager.com/ppathogens, log in, and click on the "Update My Information" link at the top of the page. Please update your user information to ensure an efficient production and billing process.

(6) LaTeX users only: Our staff will ask you to upload a TEX file in addition to the PDF before the paper can be sent to typesetting, so please carefully review our Latex Guidelines http://journals.plos.org/plospathogens/s/latex in the meantime.

Thank you again for supporting Open Access publishing; we are looking forward to publishing your work in PLOS Pathogens.

Best regards,

Italo Tempera, PHD

Academic Editor

PLOS Pathogens

Donna Neumann

Section Editor

PLOS Pathogens

Dominique Soldati-Favre

Editor-in-Chief

PLOS Pathogens

***********************************************************

Reviewer's Responses to Questions

**Part I - Summary**

Please use this section to discuss strengths/weaknesses of study, novelty/significance, general execution and scholarship.<br/><br/>

Reviewer #1: The authors have substantially improved the manuscript and have addressed nearly all of my major concerns. They performed numerous additional experiments, including analyses of acute viral replication, expanded characterization of IL-22-producing immune cell populations, additional immunological phenotyping, long-term latency studies, and mechanistic analyses of BAFF, CXCL12, and CXCL13. These new data significantly strengthen the study and support the conclusion that IL-22 promotes the establishment of chronic gammaherpesvirus infection without affecting acute viral replication.

I also appreciate that the authors have carefully moderated their conclusions throughout the manuscript. In particular, they now appropriately distinguish effects on the establishment of latency from effects on viral reactivation, acknowledge that the causal relationship between impaired germinal center responses and reduced latency cannot yet be established, and avoid overextending the implications of the MHV68 model to EBV and KSHV infection. These revisions greatly improve the scientific rigor and balance of the manuscript.

The major remaining limitation is that the cellular target and downstream mechanism of IL-22 signaling remain undefined. Although the additional BAFF data provide a plausible mechanistic link, they remain correlative and do not identify the IL-22-responsive cellular compartment responsible for the observed phenotype. However, the authors clearly acknowledge this limitation and appropriately present BAFF as a potential rather than a definitive mechanism. I believe that identifying the relevant IL-22-responsive cells and establishing causality would require a substantially larger study that is beyond the scope of the current manuscript.

Overall, I believe the authors have adequately addressed the concerns raised during the initial review, and the manuscript has been substantially strengthened. I have no further major concerns and believe the revised manuscript is suitable for publication in PLOS Pathogens.

Reviewer #2: The authors have made substantial changes to the manuscript, addressing the majority of reviewer concerns. The manuscript is greatly improved.

Reviewer #3: The authors have carefully and thoughtfully addressed each of the previous concerns.

--------------------

**Part II – Major Issues: Key Experiments Required for Acceptance**

Please use this section to detail the key new experiments or modifications of existing experiments that should be absolutely required to validate study conclusions.<br/><br/>Generally, there should be no more than 3 such required experiments or major modifications for a "Major Revision" recommendation. If more than 3 experiments are necessary to validate the study conclusions, then you are encouraged to recommend "Reject".<br/><br/>

Reviewer #1: None

Reviewer #2: (No Response)

Reviewer #3: N/A

--------------------

**Part III – Minor Issues: Editorial and Data Presentation Modifications**

Please use this section for editorial suggestions as well as relatively minor modifications of existing data that would enhance clarity.<br/><br/>

Reviewer #1: None

Reviewer #2: (No Response)

Reviewer #3: (No Response)

--------------------

PLOS authors have the option to publish the peer review history of their article (what does this mean?). If published, this will include your full peer review and any attached files.

If you choose “no”, your identity will remain anonymous but your review may still be made public.

**Do you want your identity to be public for this peer review?**  For information about this choice, including consent withdrawal, please see our Privacy Policy.

Reviewer #1: No

Reviewer #2: No

Reviewer #3: No

# Editor report: Acceptance letter

**DOI:** 10.1371/journal.ppat.1014016.r004

**Authors:** Donna M Neumann, Italo Tempera

Dear Assistant Professor Jondle,

We are delighted to inform you that your manuscript, "IL-22 promotes the establishment of chronic gammaherpesvirus infection by supporting the germinal center response," has been formally accepted for publication in PLOS Pathogens.

We have now passed your article onto the PLOS Production Department who will complete the rest of the pre-publication process. All authors will receive a confirmation email upon publication.

Thank you again for supporting open-access publishing; we are looking forward to publishing your work in PLOS Pathogens.

Best regards,

Dominique Soldati-Favre

Editor-in-Chief

PLOS Pathogens
